# A method to quantify crystallinity in amorphous metal alloys: A differential scanning calorimetry study

**DOI:** 10.1371/journal.pone.0234774

**Published:** 2020-06-22

**Authors:** Arash Yazdani, Günther W. H. Höhne, Scott T. Misture, Olivia A. Graeve

**Affiliations:** 1 Department of Mechanical and Aerospace Engineering, University of California San Diego, La Jolla, CA, United States of America; 2 University of Ulm, Ulm, Germany; 3 Kazuo Inamori School of Engineering, Alfred University, Alfred, NY, United States of America; Texas A&M University at Qatar, QATAR

## Abstract

We developed and describe a differential scanning calorimetry method for calculating the initial crystallinity, change of crystallinity and crystallinity percentage of amorphous metal alloys as a function of temperature. Using thermodynamic enthalpies of amorphous, crystalline and partially devitrified specimens, our methodology is capable of determining crystallinity percentages as low as a few percent. Moreover, the linear relationship between the set (pre-determined) and calculated crystallinities of experimental samples indicates that there is no need to prepare calibration samples before measuring the crystallinity percentage of target samples. This technique also eliminates the need for expensive *in situ* accessories, such as those required in electron microscopy. Thus, the technique is highly relevant as a primary technique for characterization of devitrification behavior in amorphous materials.

## 1. Introduction

Amorphous materials including metals, alloys, ceramics, and polymers have received a great deal of attention due to their unique properties [1–5]. For example, amorphous silicon is widely used for the fabrication of solar cells and thin film transistors. Amorphous chalcogenides are used as phase-change memory materials [6–8], biosensors [9–11], and infrared optical fibers [12–14]. For the case of amorphous metals, their high hardness, strength and wear resistance, offers the promise of creating a new class of structural materials for applications in diverse areas, including shot peening balls and fine precise polishing media [15], as well as micro gears [16]. In addition, high corrosion resistance combined with high strength and elastic modulus make bulk metallic glasses potential candidates for biomedical applications as sensors [17–18], medical implants [19–20], and self-expanding stents [21–23].

The quantity of devitrified (crystalline) phases in amorphous materials such as silica glasses and amorphous metal alloys, can drastically affect the mechanical properties [24–25]. For instance, Khanolkar *et al*. [26] studied the shock wave response of partially devitrified Fe_49.7_Cr_17.7_Mn_1.9_Mo_7.4_W_1.6_B_15.2_C_3.8_Si_2.4_ and reported that the presence of a small quantity of crystalline phases within the amorphous matrix results in an outstanding elastic limit. Magnetic [27–29] and corrosion [30–31] properties of amorphous materials are also influenced by their crystallization behavior. Zhukova *et al*. [28] explored the effect of stress annealing on magnetic properties and high frequency impedance of Fe_75_B_9_Si_12_C_4_ amorphous glass-coated microwires. The annealing treatment significantly decreased the coercivity and modified the hysteresis behavior. Additionally, the coercivity, remanent magnetization, and magnetoimpedance effect of microwires was altered by changing time and temperature during the stress-annealing process. Zhou *et al*. [32] studied the effect of crystallinity percent on corrosion behavior of Mg_65_Cu_25_Y_10_ and Mg_70_Zn_25_Ca_5_ bulk metallic glasses and reported that fully amorphous BMGs possess a lower *i*_corr_ and more noble *E*_corr_ compared to their crystalline counterparts.

In order to quantify the level of devitrification of amorphous materials, many techniques have been developed, including X-ray diffraction (XRD), electron backscatter diffraction (EBSD), and thermal analysis routes such as differential scanning calorimetry (DSC) [33–34]. Among these methods, XRD is the most extensively used, following two major approaches for quantification, either employing individual peaks in the XRD pattern or employing the entire pattern to define a relationship between the phase composition and the intensity and/or cumulative area of peaks. As an example of the former category, Hermans and Weidinger [35–36] developed a procedure for crystallinity quantification of polymers by XRD based on three assumptions: (i) it should be possible to measure the crystalline intensity (*I*_c_) and amorphous intensity (*I*_a_) of samples with varying crystallinity percentages, (ii) there is a proportionality between the experimentally measured crystalline intensity and the crystalline fraction (*X*_c_) in the sample, and (iii) there is a proportionality between the experimentally measured amorphous intensity and the amorphous fraction (*X*_a_) in the sample. Thus,

$$X_{c}=p\;I_{c}$$ (1)

$$X_{a}=q\;I_{a}$$ (2)

$$X=X_{a}+X_{c}$$ (3)

where *p* and *q* are proportionality constants. Combining these equations, one obtains:

$$q\;I_{a}=X-p\;I_{c}$$ (4)

and

$$I_{a}=X/q-p\;I_{c}/q$$ (5)

The values of *I*_a_ and *I*_c_ can be determined for samples with different crystallinity percentages. Plotting *I*_a_ versus *I*_c_ should result in a straight line whose slope is *p*/*q*. The intercepts on the *x* and *y* axes provide the intensity values of 100% crystalline and 100% amorphous samples, respectively. The crystallinity percentage, *X*_cr_, is given by:

$$X_{cr}=\frac{X_{c}\cdot 100}{X}=\frac{I_{c}\cdot 100}{I_{c}+\frac{q\cdot I_{a}}{p}}$$ (6)

Although this method is widely used, it has major limitations. For samples with preferred grain orientation, the peak intensity for a specific direction is intensified leading to significant errors in the calculation. In powders, this effect could be limited by reducing the size by grinding. However, the process of grinding can cause other unwanted effects, such as polymorphic transformations or devitrification of amorphous samples. The powders should be small enough to yield reproducible diffracted intensities, but not too small since reduced crystallite size can lead to broadening of the XRD peaks [37–38]. Also, determination of crystalline (*I*_c_) and amorphous (*I*_a_) intensities from experimentally recorded XRD patterns can be difficult because it depends on a potentially subjective determination of the baseline intensity of the pattern due to thermal and air scattering [36, 38]. Additionally, generating a calibration curve requires preparing samples with various crystallinity percentages, which is accomplished by mixing appropriate proportions of crystalline and amorphous standard phases. Here, heterogenous mixing and sampling can have an effect on the results, especially when preparing mixtures of extreme compositions (either very dilute or concentrated) [39]. Inhomogeneity is probably the greatest source of error for many quantitative methods, especially with crystalline and amorphous components where the bulk density difference between the two is significantly large. Problems can arise in the calibration and validation samples, as well as samples with active ingredients [40].

Nunes *et al*. [39] proposed that gradual crystallization of an amorphous component, usually through heating, is similar to characterizing a series of physical mixtures prepared with increasing percentage of crystalline component. In other words, the *in situ* crystallization method can circumvent the problem of preparing solid mixtures containing very small amounts of either crystalline or amorphous components. Generally, this technique requires using an XRD unit equipped with a hot-stage for heating and suffers from the limited time resolution and sensitivity leading to a lack of detection of subtle changes in the crystallinity and, as mentioned earlier, the presence of an amorphous phase makes it quite challenging to precisely define the baseline in XRD patterns. In order to quantify the content of crystalline phases, XRD peaks corresponding to such phases need to be identified and labeled properly. This could be problematic for systems with phases whose peaks overlap. For the case in which one peak is assigned to more than one phase, there is always the possibility that phase identification and peak deconvolution steps cannot be implemented properly. In general, conventional XRD techniques are less accurate for characterization of nanostructured materials and samples containing amorphous phases, since diffraction patterns become significantly broader and crystalline features may be less discernible.

Quantification of XRD patterns could be improved by implementing whole pattern methods. One of these commonly used techniques is Rietveld refinement, by which all reflections are simulated by calibrated crystallographic parameters [41]. However, issues such as baseline definition can result in lack of certainty over the applicability as well as accuracy of this method. Transmission electron microscopy (TEM) can be helpful in determining crystalline phases by utilizing the electron diffraction feature of this technique. Nevertheless, sample preparation procedures are complicated, and analysis is not always straightforward, especially for fully unknown samples. Additionally, drawing a solid and reliable conclusion requires obtaining and analyzing a great many images to make sure that the results are not local. Electron backscatter diffraction (EBSD) is used for orientation determination, as well as phase identification. In this method, the EBSD pattern is generated from the diffraction of the electron beam interacting with crystallographic planes within the specimen [42], which requires accurate surface polishing and finishing of bulk samples. In order to obtain EBSD patterns of high quality and with a high indexing success rate, surface damage typically introduced during polishing should be minimized. This could be fulfilled by ion etching, using either a broad or focused beam, which can be expensive and time-consuming [43–44]. Also, in order to index the recorded pattern and distinguish between amorphous and crystalline regions, a comprehensive knowledge of the crystal structure of devitrified phases is required. Apart from the limitations that have been highlighted for each of the methods described above, most techniques collect signals from a small region of the sample, resulting in a bias from the extreme locality of the signal. For instance, EBSD patterns are generated from the signals coming from a small interaction volume at the surface of the sample with a penetration depth of typically less than 50–100 nm [42]. This issue is attenuated when the XRD method is utilized.

DSC is superior to most other common methods used for quantifying the crystallization process of amorphous materials. The technique can partially overcome the majority of the limitations described previously. Nevertheless, most DSC studies associated with the determination of change of crystallinity of amorphous systems, have been mainly focused on exploring the crystallization of polymers. In order to calculate the crystallinity percentage of a polymeric sample, the specific heat of fusion for a partially crystalline polymer is measured from the area under the endothermic peak in the heat flow curve and then divided by specific heat of fusion for a fully crystalline sample. The crystallinity percentage obtained from this method, however, is an average value which is valid for the temperature region around the melting point. In other words, the change of crystallinity that takes place upon heating the sample from room temperature to the melting point is not considered. In order to obtain an accurate temperature dependence of crystallinity fraction [*W*_c_ (T)] for a polymer, variation of enthalpy as a function of temperature for the sample of unknown crystallinity should be calculated and then compared using the following equation:

$$W_{c}\left(T\right)=\frac{h_{am}\left(T\right)-h_{s}\left(T\right)}{h_{am}\left(T\right)-h_{cryst}\left(T\right)}$$ (7)

where *h*_am_, *h*_s_, and *h*_cryst_ are the specific enthalpies of a fully amorphous sample, the test sample, and a fully crystalline sample, respectively. The specific enthalpy of the test sample (*h*_s_) relative to a reference state at an initial temperature, *T*_ini_, can be calculated from the specific heat capacity, *C*_p_(*T*), as follows:

$$h_{s}\left(T\right)-h_{s}\left(T_{ini}\right)=\int_{T_{ini}}^{T}C_{p}\left(T\right)dT$$ (8)

**Fig 1:**
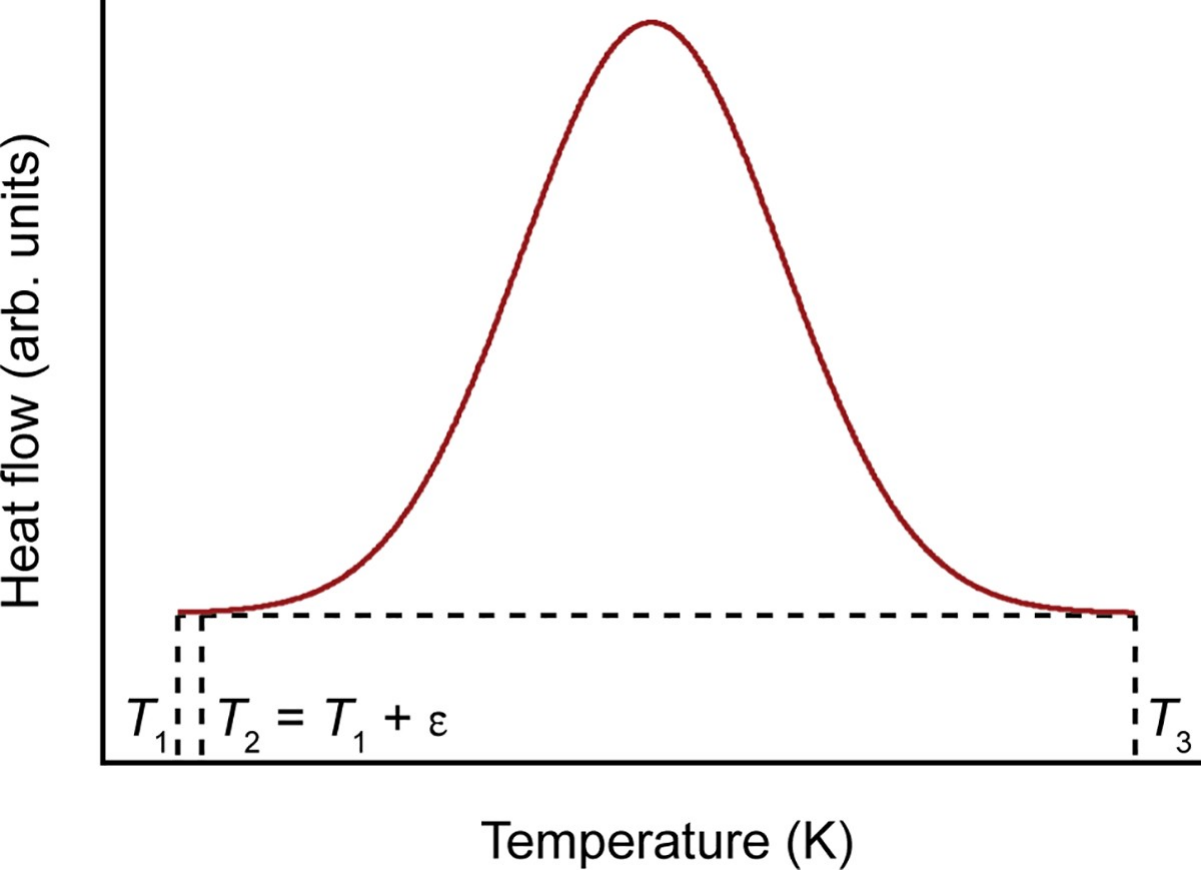
Schematic representation of an exothermic crystallization peak. *T*_1_ and *T*_3_ represent the temperatures at which crystallization starts and ends, respectively.

Measuring the specific heat capacity is a well-known isothermal process in which the heat flow of an empty pan (baseline) is subtracted from that of the test sample. The result is then divided by the mass of the test sample and heating rate to obtain specific heat capacity as a function of temperature. Details of this methodology are described by Höhne *et al*. [33]. This method has yielded the most accurate results compared to other approaches in polymers. However, there is no report that has extended this technique for non-polymeric systems such as amorphous alloys and silica glasses. Some researchers have attempted to quantify the crystallization process of Fe-based metallic glasses [45–46] using an isochronal DSC experiment. This approach was introduced by Johnson-Mehl-Avrami in order to study the kinetics of isothermal solid-state phase transformations (here glass to crystal) [47–48]. They postulated that the fraction of crystallized component is proportional to the amount of heat evolved during crystallization, which is proportional to the area under the exothermic peak associated with the crystallization [49]. The thermal evolution of the crystallized fraction, α(*T*), can then be calculated as a function of temperature for different heating rates using:

$$\alpha \left(T\right)=\frac{\int_{T_{o}}^{T}\left(\frac{dh}{dT}\right)dT}{\int_{T_{o}}^{T_{\infty }}\left(\frac{dh}{dT}\right)dT}=\frac{A_{T}}{A}$$ (9)

where *T*_o_ and *T*_∞_ correspond to the temperatures of onset and end of crystallization, d*h*/d*T* is the heat capacity at a constant pressure, A_*T*_ is the area under the DSC curve between the onset temperature and a given temperature, and A is the area under the curve between onset and end of crystallization [49]. Basically, partial integration of the heat flow curve versus temperature curve results in the heat of crystallization versus temperature function. The integration method results in a “change of crystallinity” rather than “crystallized fraction” as a function of temperature. Given Eq 9, the initial crystallinity for a sample is an unknown and cannot be determined. Fig 1 shows a typical DSC crystallization peak of an arbitrary sample with an unknown initial crystallinity. Crystallization starts at *T*_1_ and ends at *T*_3_. The area under the peak between *T*_1_ and *T*_3_ equals A. Assuming that during heating the sample does not undergo crystallization until it reaches *T*_1_, the initial crystallinity could be calculated by dividing the area under the curve between *T*_1_ and *T*_2_ = *T*_1_ + ε by the area under the curve between *T*_1_ and *T*_3_.

**Fig 2:**
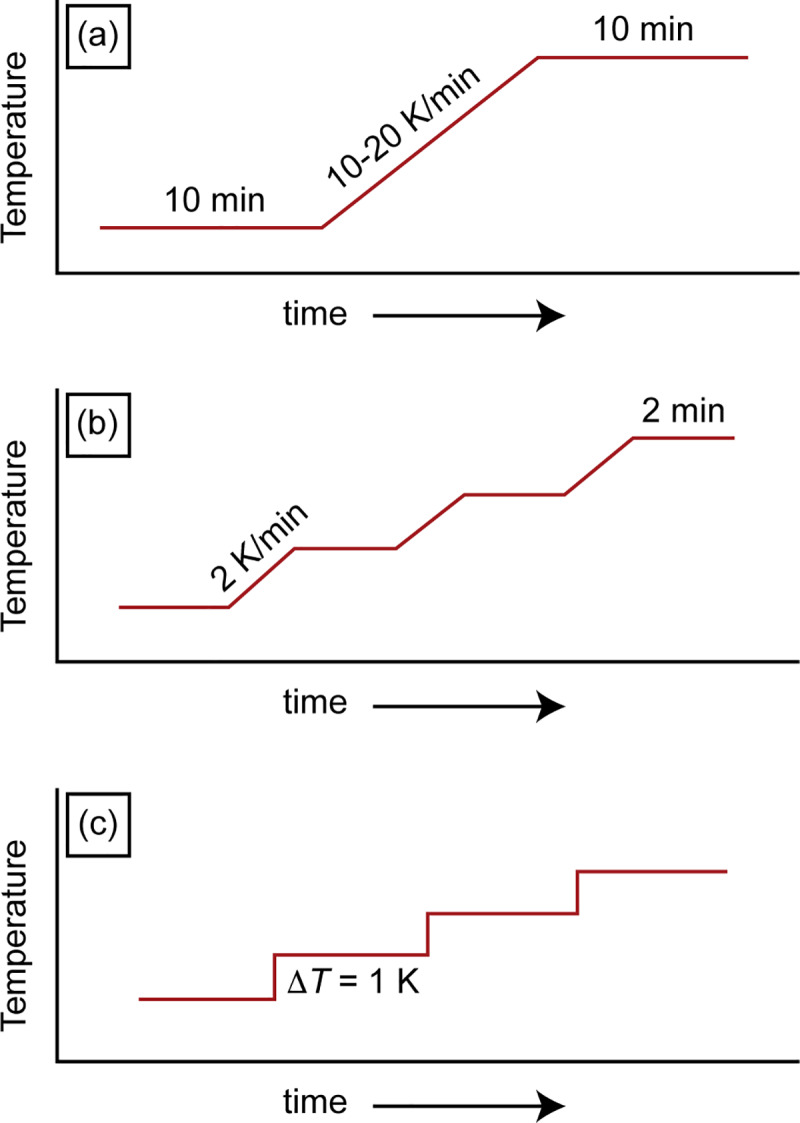
(a) Dynamic, (b) isostep and (c) area methods for measuring the specific heat capacity of a material using differential scanning calorimetry. (a) The dynamic method has an isothermal segment followed by heating with a constant and high heating rate (10–20 K/min) until the final isothermal segment. (b) The isostep method consists of short dynamic stages along the whole temperature range with isothermal stages before and after each heating segment. (c) The area method consists of consecutive isothermal segments with no heating stages in between [54].

Different modes of DSC have been attempted to obtain the most reliable and accurate *C*_p_ values. Conventional DSC using a dynamic temperature program is one of the most common techniques. It consists of three segments, starts and ends with two 10- to 15-minute isothermal stabilization segments, between which a linear temperature ramp with a constant and high heating rate (10–20 K/min) is implemented [33]. In the isothermal mode, achieved by a temperature-modulated DSC (TM-DSC), there are short dynamic stages along the entire temperature range with isothermal stages before and after each heating segment to stabilize the temperature. The temperature increase at each heating stage needs to be small, between 1 and 3 K. Since the temperature jumps are small, the isothermal stages could be as short as 2–3 minutes ensuring thermal equilibrium of the sample [50]. Sauerbrunn *et al*. [51] claimed that temperature modulated DSC has advantages over conventional DSC for determination of heat capacity and crystallinity. However, Schawe and Hess [50] proved that the method to determine the initial crystallinity by TM-DSC does not have any advantage compared to the classical evaluation methods using conventional DSC. The claimed apparent superiority of TM-DSC over conventional DSC is simply based on a poor baseline selection. Also, the complex heating program and evaluation procedure of TM-DSC could create additional errors in the enthalpy determination [50]. Roura *et al*. [52] measured the small *C*_p_ differences between the amorphous and crystalline phases of silicon using four different approaches, namely conventional and temperature-modulated DSC at a constant heating rate and with isotherms. They concluded that conventional DSC with isotherms results in the best reproducibility for the measurement of *C*_p_ variations due to crystallization. Ferrer *et al*. [53] measured the specific heat capacity of three materials including slate, water and potassium nitrate by DSC through three different heating programs, dynamic, isostep and area methods, as described in Fig 2. They found relative errors smaller than 3% using the area method, while the dynamic and isostep techniques yielded values with errors up to 6% and 16%, respectively. As a consequence of high heating rates applied in the dynamic and isostep techniques, there was an abrupt temperature change at the initial and end points. Hence, DSC temperature sensors were not able to react fast enough to read and record the real temperature. Consequently, there was noise at the beginning and end of each heating segment leading to a lower measurement sensitivity.

In response to the issues described above, we present a DSC technique that can quantify the amount of devitrified phase in amorphous metal alloys over a broad range of temperatures. The method results in both the change of crystallinity and crystallized fraction (or crystallinity percentage) as a function of temperature of Fe-based amorphous powders using a conventional DSC with a dynamic temperature program. Specific heat capacity is calculated from the heat flow curve through the integration of *C*_p_, then an enthalpy function is obtained from which the change of crystallinity and crystallinity percentage are determined.

## 2. Experimental methodology

Amorphous SAM2×5 with a chemical composition of Fe_49.7_Cr_17.7_Mn_1.9_Mo_7.4_W_1.6_B_15.2_C_3.8_Si_2.4_ was used as a starting material [54–58]. To fully crystallize the amorphous SAM powder, a heat treatment to a temperature of 1323 K was implemented using a heating rate of 30 K/min in a Lindberg 59744-A tube furnace. The heat treatment time was 2 hours under a high purity N_2_ atmosphere (99.999%). The powder was subsequently cooled to room temperature in the protected N_2_ atmosphere.

X-ray diffraction (XRD) patterns at room temperature were obtained using a D2 Phaser (Bruker AXS, Madison, WI) using a step size of 0.014 degrees 2θ, Cu Kα radiation, and a count time of 3 s by scanning from 30 to 90 degrees 2θ. In order to determine the devitrified phases during the crystallization process, high temperature *in situ* XRD experiments were also completed in high-purity helium and 4% hydrogen in nitrogen on a D8 Advance system (Bruker AXS, Madison, WI) with an Anton Paar HTK 1200N furnace and Cu anode X-ray tube. Heating rate was set to 30 K/min for heating between scans. Hold (scanning) time for each measurement was 30 minutes. Scan step was 0.03° and count time was 1 s/step, which corresponds to a scanning rate of 1.54°/minute.

Thermal analyses were carried out by differential scanning calorimetry on a SDT Q600 (TA Instruments, New Castle, DE) instrument using a heating rate of 30 K/min to a temperature of 1273 K in flowing argon. Alumina crucibles of 90 μL capacity were used. All alumina crucibles were taken through a homogenization process, consisting of heating and cooling under the same conditions as the SAM2×5 powders in the DSC instrument. For the DSC experiments, approximately 100 mg of powder were loaded into an alumina crucible and an empty alumina crucible was used as a reference. Powder samples with five different crystallinity percentages were prepared by mixing the appropriate amounts of amorphous and crystalline SAM2×5 powders. For each crystallinity percentage, three independent samples were prepared and tested using DSC. The amounts are tabulated in Table 1. Contrary to most conventional characterization techniques, the sample preparation for DSC experiments is not sensitive to the need for homogeneous mixing, since the heat flow measured by DSC is acquired from the whole material mass inside the crucible regardless of how homogenous it has been mixed and prepared. This could be considered one of the advantages of the DSC method.

**Table 1:** Nominal crystallinity, mass of individual components, and average crystallinities of powder batches prepared by mixing amorphous powders and crystalline powders.

Sample	Nominal crystallinity (%)	Mass of amorphous powder (mg)	Mass of crystalline powder (mg)	Average crystallinity (%) ± standard deviation
SAM5%	5	95.0	5.1	5.1 ± 0.1
		95.1	5.2	
		94.9	4.9	
SAM20%	20	80.2	20.0	20.0 ± 0.1
		80.1	19.8	
		80.1	19.9	
SAM40%	40	60.2	40.0	40.0 ± 0.2
		59.9	40.3	
		60.0	39.8	
SAM60%	60	40.1	59.9	60.0 ± 0.1
		39.8	59.9	
		40.2	60.1	
SAM80%	80	20.2	80.1	80.0 ± 0.1
		19.9	79.9	
		19.9	80.2	

The heating program consisted of an isothermal heating at 323 K for 30 minutes, followed by heating from 323 K to 1208 K with a heating rate of 30 K/min, and finally another isothermal segment at 1208 K for 30 minutes. Isothermal dwell times of 30 minutes guarantees the thermal equilibrium of the sample. Additionally, a maximum temperature of 1208 K was selected because it lies between the completion of crystallization and the melting temperature (about 1473 K) of the SAM2×5 material. Using this temperature program, heat flow curves for the empty crucibles and the test samples were recorded under a high purity Ar atmosphere (99.999%). In order to obtain reliable and accurate results, system calibrations were completed. The TGA weight calibration made use of alumina standard specimens and the DSC heat flow calibration made use of sapphire standard specimens. After calibration, testing was completed on an empty crucible, followed by testing of an amorphous powder loaded into the same alumina crucible. This cycle, including system calibration, was repeated for the crystalline and partially devitrified samples.

## 3. Results and discussion

**Fig 3:**
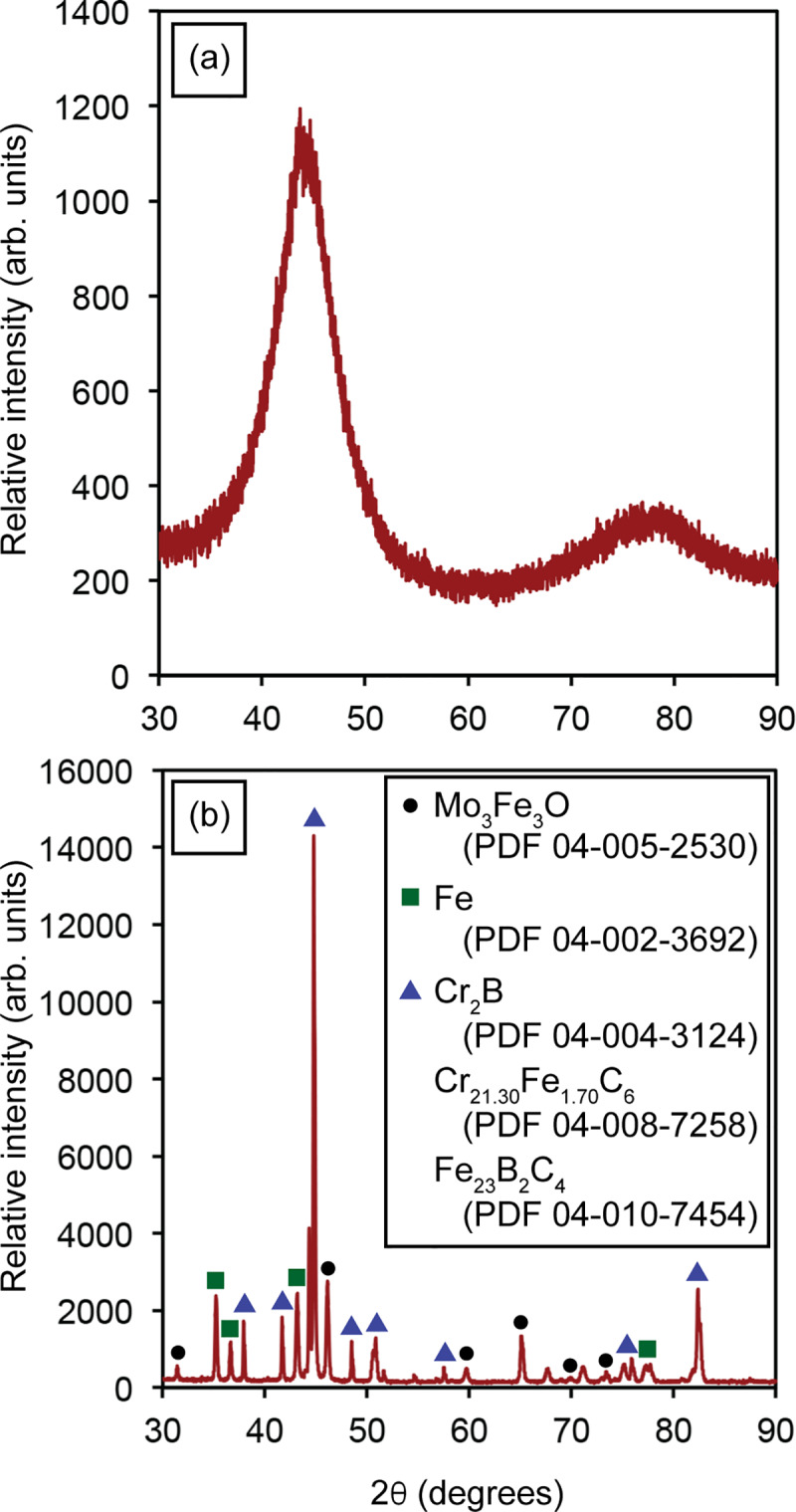
X-ray diffraction patterns of (a) amorphous and (b) crystalline SAM2×5 powders. The crystalline powders were obtained by heat-treating the amorphous powders at 1323 K for 2 hours under nitrogen atmosphere.

**Fig 4:**
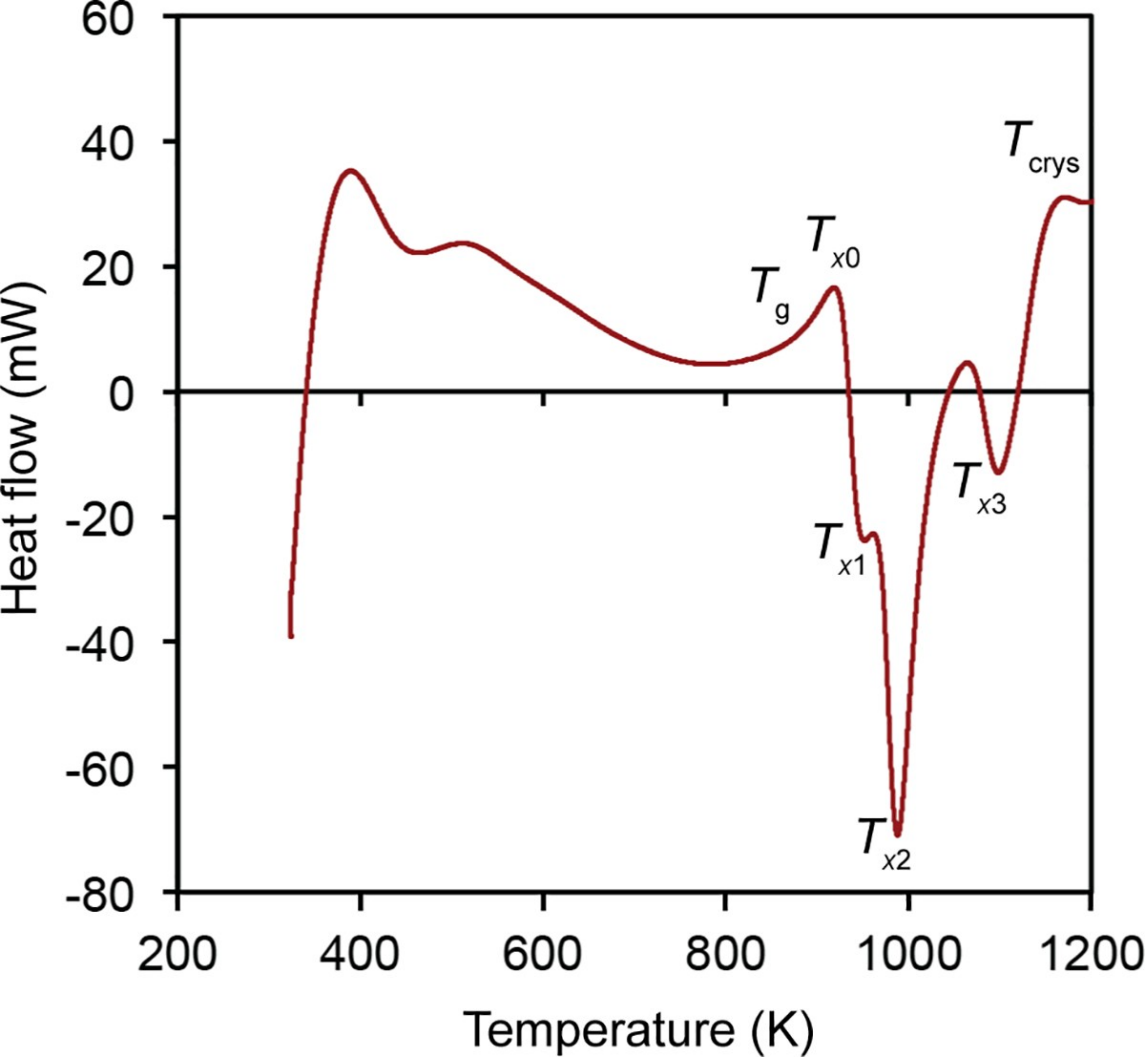
Heat flow curve of amorphous SAM2×5 powder obtained using a heating rate of 30 K/min in an argon atmosphere.

Fig 3 illustrates the XRD patterns of the amorphous and crystalline SAM powders. The amorphous SAM powder exhibits the typical broad pattern confirming its amorphous nature. The crystalline SAM powder exhibits sharp peaks belonging to carbides, borides, oxides and metallic iron. The SAM2×5 amorphous powder was initially characterized using DSC (Fig 4) resulting in a glass transition temperature (*T*_g_) of ~883 K. The crystallization process initiated at *T*_*x*0_ = 918 K followed by three additional crystallization events at *T*_*x*1_ = 953 K, *T*_*x*2_ = 988 K, and *T*_*x*3_ = 1098 K. Finally, SAM2×5 achieved full crystallinity at *T*_crys_ = 1173 K.

**Fig 5:**
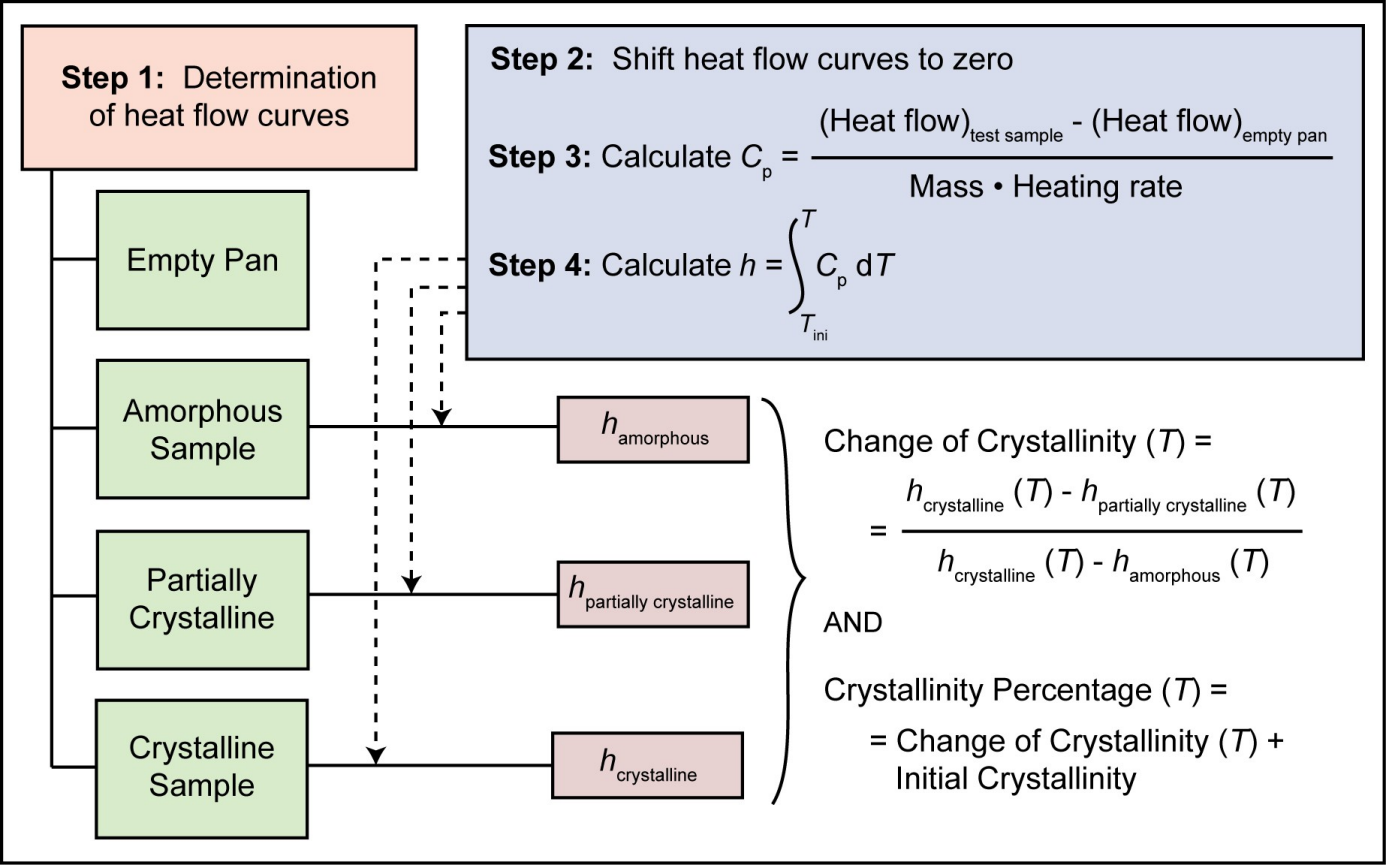
A schematic representation of our DSC-based methodology for determination of the change of crystallinity and crystallinity percentage as a function of temperature.

**Fig 6:**
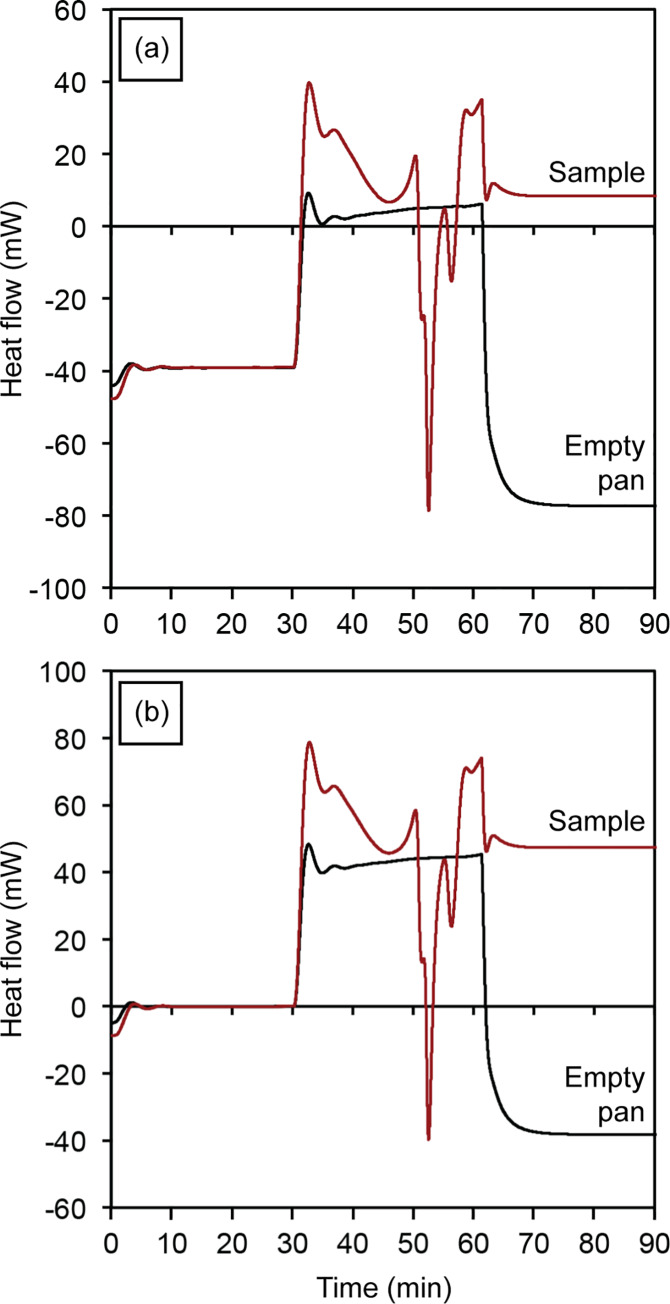
Heat flow curves with respect to time for an empty alumina pan and the same pan loaded with powder test sample (a) as-recorded by differential scanning calorimetry and (b) after shifting upwards so that the initial isothermal segments start at zero heat flow.

Fig 5 describes the methodology used in this study. In the first step, heat flow curves are determined for an empty crucible, an amorphous sample, a partially crystalline sample, and a fully crystalline sample [33]. Then, the change of crystallinity of a sample is calculated based on the change in enthalpy as a function of temperature, which is derived by the integration of the specific heat capacity curve using Eq 8. For this purpose, using the heating program described in the experimental methodology, the heat flow of an empty alumina crucible is first recorded with respect to time. In a second experiment, the same crucible is loaded with powder test sample and recorded [Fig 6(A)]. In both cases, an empty alumina crucible is used as a reference. Then, the initial isothermal segment of the heat flow curves of both the empty alumina crucible and test sample are shifted upwards or downwards for an initial heat flow of zero, as illustrated in Fig 6(B). Incorporating isothermal segments into the beginning and end of the heating program along with plotting the heat flow curves as a function of time helps to ensure that the specimen experiences equilibrium conditions during the isothermal heating step, which can be distinguished by a flat (constant) heat flow during the isothermal segments. Moreover, these flat curves at the beginning and end of the heating program makes it easier and more accurate to do further adjustments such as shifting the curves either upwards or downwards. For the next steps in the analysis, we will determine temperatures at which specific thermal events—such as structural relaxation, glass transition, and crystallization—take place.

**Fig 7:**
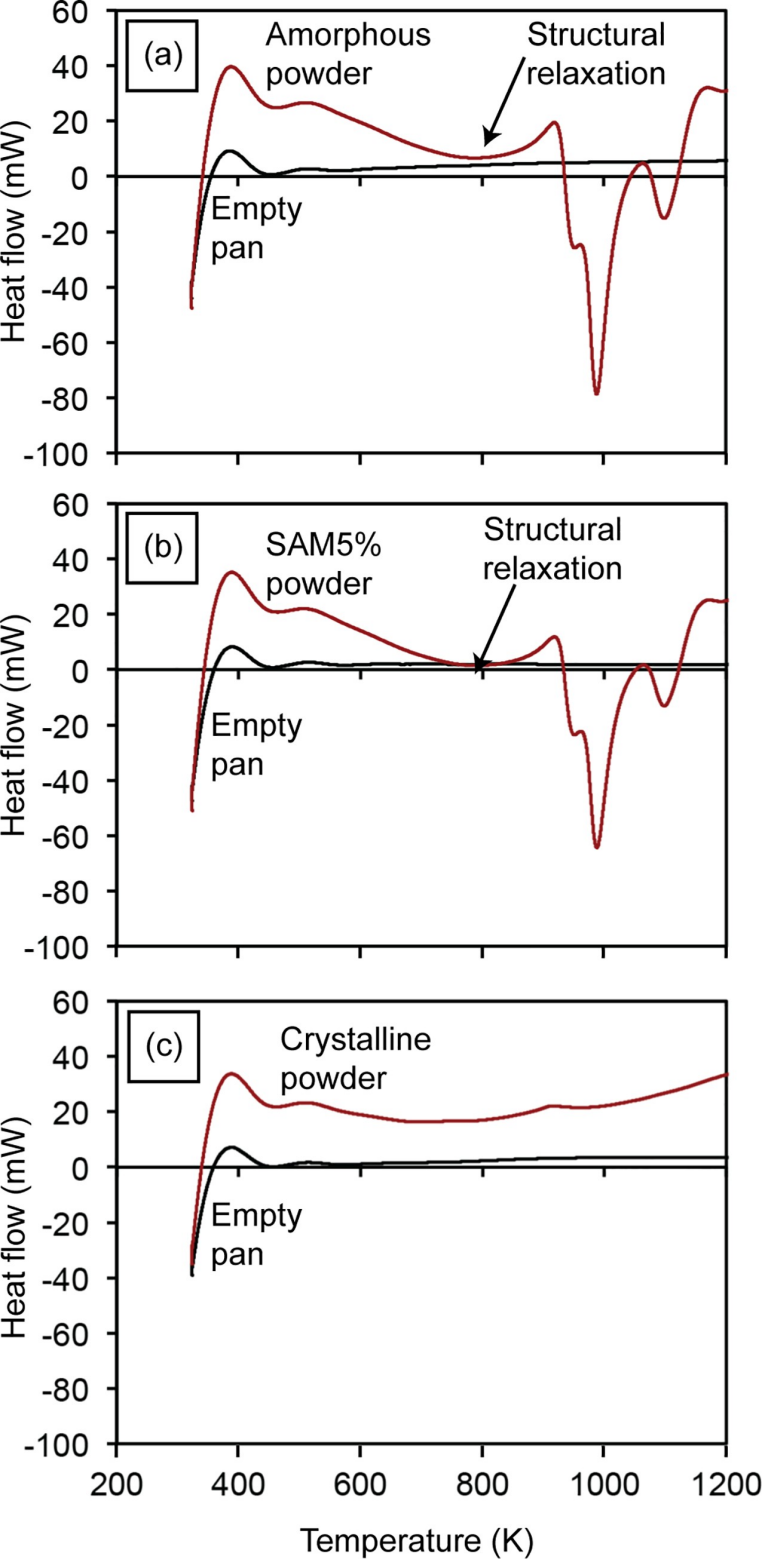
Heat flow curves with respect to temperature of (a) empty pan and amorphous SAM2×5 powder, (b) empty pan and SAM5% powder mixture with a known initial crystallinity, and (c) empty pan and crystalline SAM2×5 powder. Downward peaks in these charts represent exothermic events.

Fig 7 illustrates typical heat flow curves recorded for the empty pan, the amorphous SAM2×5 powder, the powder mixture of SAM5%, and the crystalline SAM2×5 powder with respect to temperature. Crystallization of the amorphous SAM2×5 powder takes place at temperatures between 918 K and 1173 K [Figs 4 and 7(A)]. The exothermic crystallization peaks shrink slightly for sample SAM5% [Fig 7(B)]. The crystallization peak in amorphous metallic systems is preceded by another exothermic event that spans a wide temperature range from approximately 600 to 900 K, as seen in the graphs of Fig 7(A) and 7(B). This exothermic event represents the structural relaxation of the amorphous phase whose existence, albeit minimal, in the fully crystalline SAM powders [Fig 7(C)] could be due to the existence of a very small content of amorphous phase in this powder.

**Fig 8:**
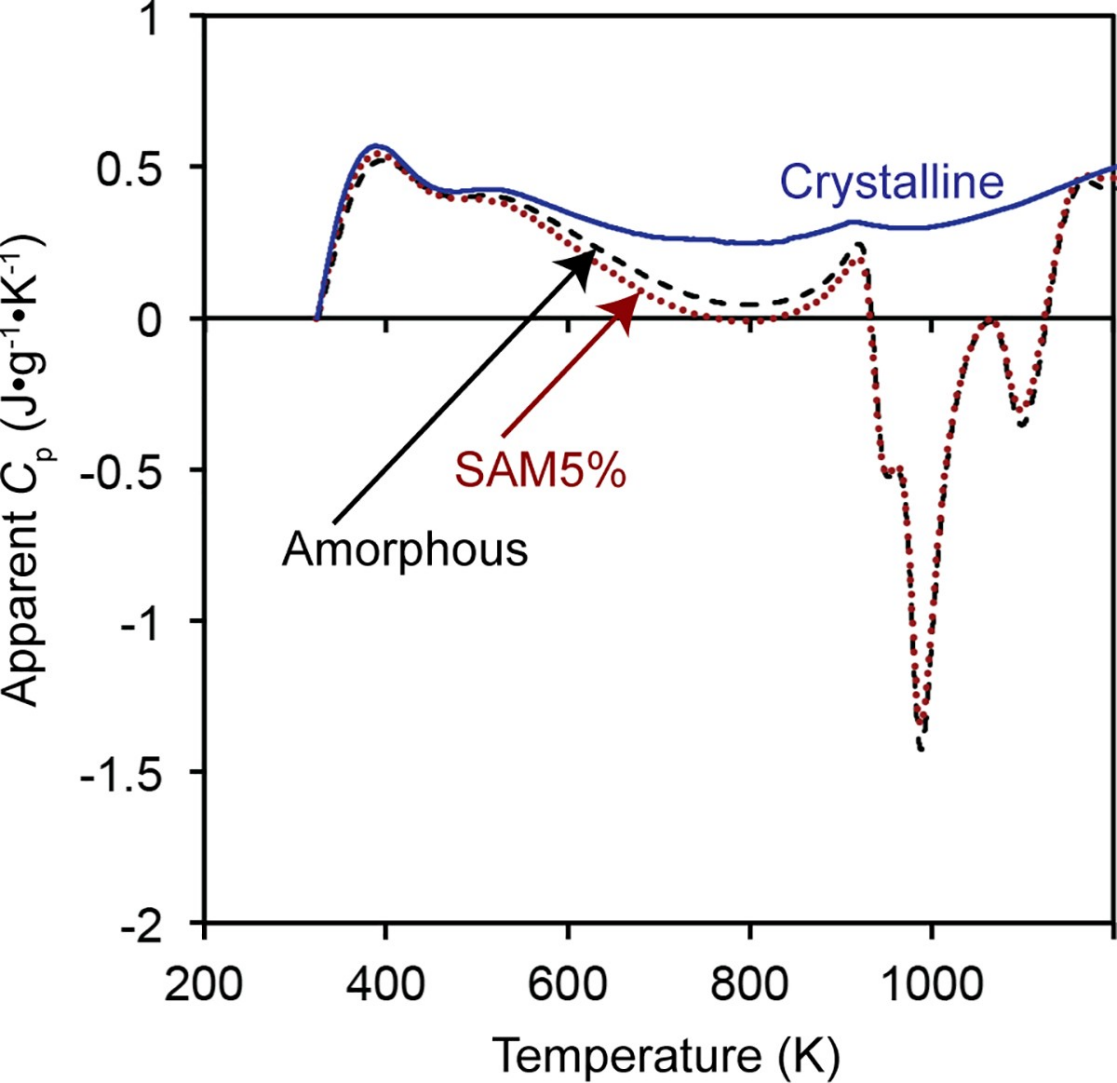
Apparent heat capacity, *C*_p_, of amorphous, SAM5%, and crystalline powders as a function of temperature.

**Fig 9:**
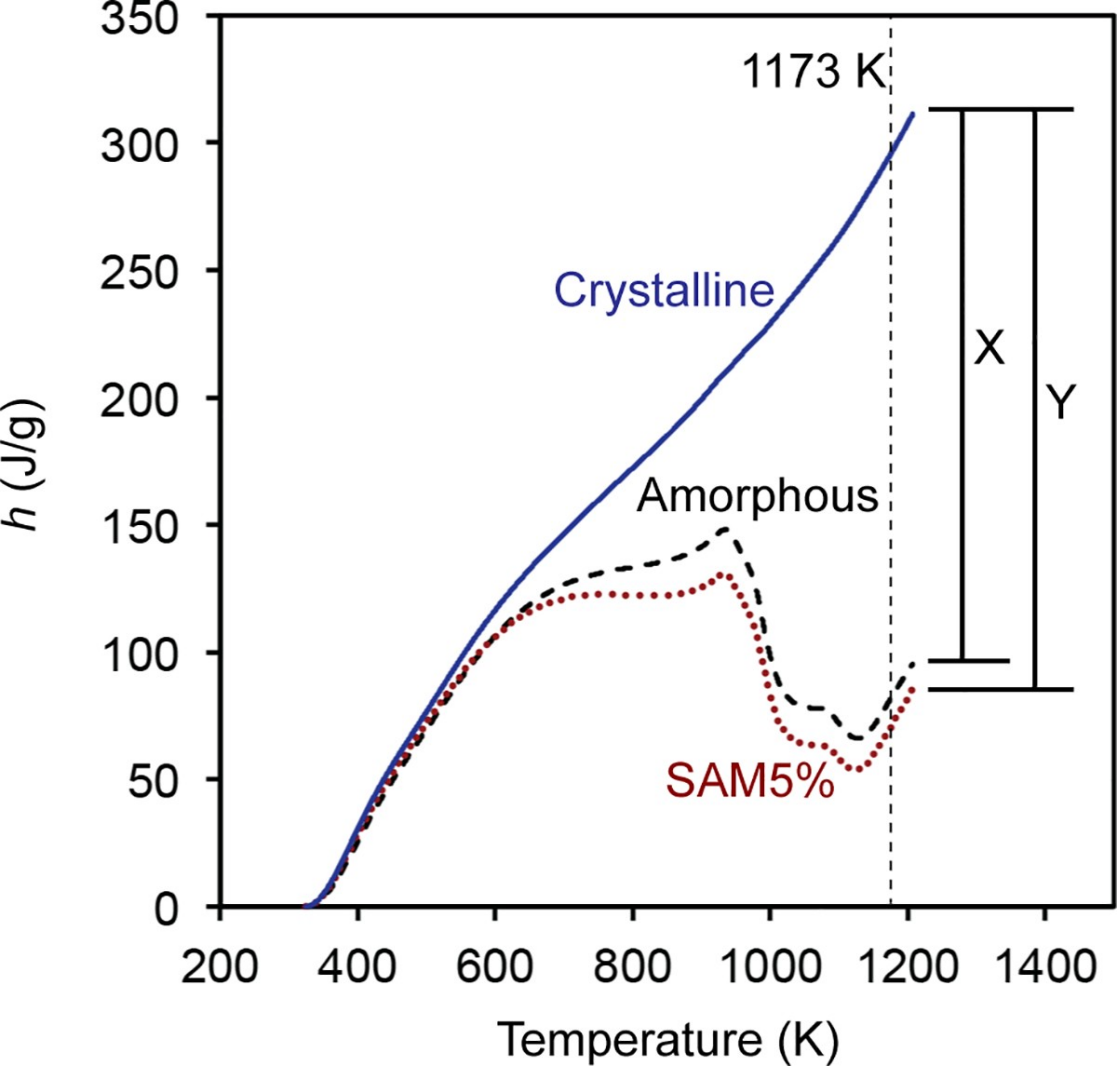
Enthalpy of amorphous, SAM5%, and crystalline powders as a function of temperature. The difference between the enthalpy curves of the amorphous and crystalline powder samples at temperatures above 1173 K (shown by an X) represents the enthalpy difference between amorphous and crystalline states, which is the crystallization enthalpy of amorphous SAM2×5 and is a constant value. The difference between the enthalpy curves of SAM5% and crystalline powder at any arbitrary temperature (shown by a Y) yields the crystallization enthalpy of SAM5% at each specific temperature. Therefore, Y is a function of temperature and becomes larger as temperature is increased.

In the next step, the heat flow of the empty crucible is subtracted from that of the crucible containing the test powders, which results in the heat flow through the powder sample. This value is divided by the mass of the test powders and heating rate to obtain the apparent heat capacity of the powder sample. Fig 8 illustrates the apparent heat capacity curves for the three samples. The *C*_p_ value of the crystalline SAM2×5 powder is about 0.5 J/g•K, which is in good agreement with that of steels [59]. Subsequently, the variation of enthalpy for each sample is calculated using Eq 8 by integration of the apparent heat capacity curves. These calculations result in curves illustrated in Fig 9. For the sake of simplicity in the calculation, the initial enthalpy of each sample is assumed to be zero. The initial enthalpy can be any arbitrary value since the change of enthalpy is taken into consideration in subsequent calculations. Above 1173 K, where the crystallization process ends, the *h* curves of all samples become parallel and linearly increase with an increase of temperature. The difference between the enthalpy of the SAM5% sample with that of the crystalline sample is proportional to the temperature-dependent change of crystallinity of the SAM5% sample.

**Fig 10:**
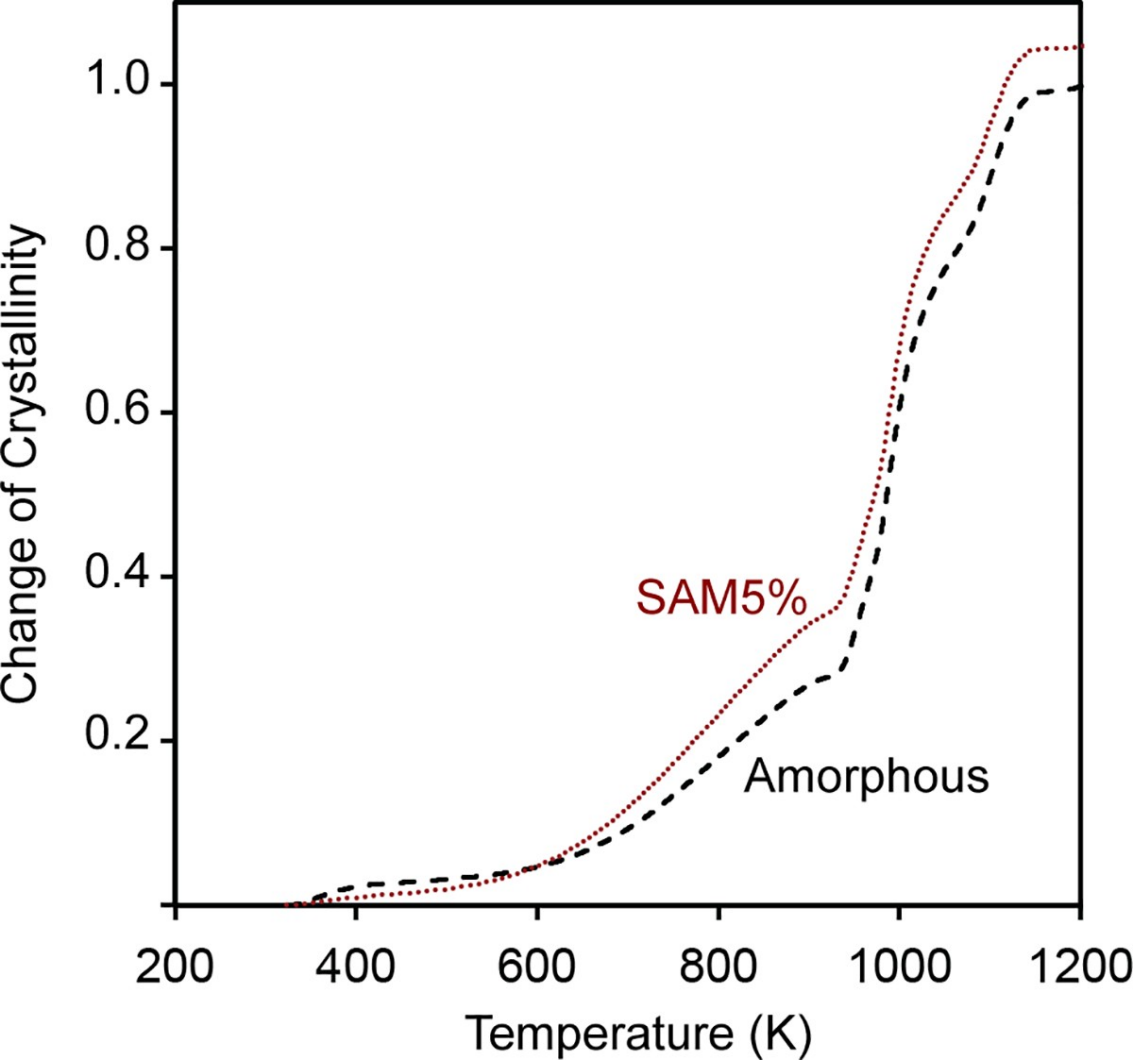
Change of crystallinity as a function of temperature for amorphous and SAM5% powders as a function of temperature without correction for structural relaxation.

The difference between the enthalpy curves of the amorphous and crystalline powder samples at a temperature above 1173 K (shown by an X in Fig 9) represents the enthalpy difference between amorphous and crystalline states, which is basically the crystallization enthalpy of amorphous SAM2×5 and is a constant value. On the other hand, the difference between the enthalpy curves of SAM5% and crystalline powder at any arbitrary temperature (shown by a Y in Fig 9) yields the enthalpy of SAM5% at each temperature across the curve. The line representing Y is moved from left to right at each point and used to obtain a value at each temperature. Therefore, Y is a function of temperature. Dividing the enthalpy difference between SAM5% and crystalline SAM2×5 (Y in Fig 9) by the difference between the enthalpy curves of the amorphous and crystalline powder samples at temperatures above 1173 K (X in Fig 9), yields the change of crystallinity as a function of temperature, as illustrated in Fig 10. One can see that in Fig 9, the magnitude of X at 1173 K, which represents the crystallization enthalpy of amorphous SAM2×5, is smaller than that of Y, which is the crystallization enthalpy of partially devitrified SAM5%. Unfortunately, this will cause the change of crystallinity of SAM5% to be greater than that of the amorphous SAM2×5 powder (Fig 10), an unexpected situation since one would expect the amorphous sample to have a greater change in crystallinity as the devitrification process evolves. Such abnormalities were not observed for other test samples with crystallinity percentages of 20, 40, 60, and 80%. The reason for this effect is that SAM5%, due to its very low crystallinity, has a crystallization enthalpy close to that of amorphous SAM2×5 and natural variability in the experiments results in this issue. As will be discussed later, we developed a procedure for correcting enthalpy curves that resolves this. Also, as seen in Fig 10, the crystallization process for both samples starts at a temperature between 473 K and 573 K, gradually increases until 923 K, and is followed by a more rapid crystallization process ending around 1173 K.

According to previous results [60–64], there is no crystallization event below the *T*_g_ of amorphous materials. The degree of disorder in amorphous metals partially depends on the occurrence of short-range order (SRO), which involves changes of the local order such as rearrangement of neighboring atoms and local bond lengths. Annealing a metallic glass below the glass transition temperature can disturb the SRO. This process is known as structural relaxation. Van Den Beuker and Radelaar [64] proposed a model for short range order that considers two different processes: (i) chemical short range order (CSRO) which is associated with redistribution of the constituent atoms, known to be reversible and is more or less analogous to short range order in crystalline alloys [61, 63] and (ii) topological short range order (TSRO) which is connected to topological changes, decreased atomic spacing and change of material volume, and generally attributed to irreversible relaxation [61, 63]. At lower temperatures, the more ordered states are favored while at higher temperatures the more disordered states dominate. Given that true equilibrium takes place when the crystalline state is reached, CSRO and TSRO processes independently relax towards the equilibrium states [64]. What takes place below the glass transition temperature is, in fact, structural relaxation phenomena that eventually results in crystallization starting at *T*_g_. In other words, below the glass transition temperature we are facing densification of the glass (accompanied by a decrease in enthalpy) and possibly formation of nano-sized nuclei that enable the crystallization process above *T*_g_ (cold crystallization) [65–67]. Also, there are many reports that associate the exothermic reactions just before the glass transition to the annihilation of free volume and structural relaxation [68–71]. Fig 7 shows this feature in the heat flow curves of the amorphous, SAM5%, and crystalline samples. In order to make sure that final crystallinity values are not affected by the structural relaxation effect, regardless of its origin, the enthalpy curves for powders need to be corrected. For this purpose, as will be discussed in detail later, we can safely ignore the sections of the enthalpy curves below the temperature of crystallization. Here, we disregard the enthalpy change below 898 K, which is slightly below the temperature of crystallization onset.

**Fig 11:**
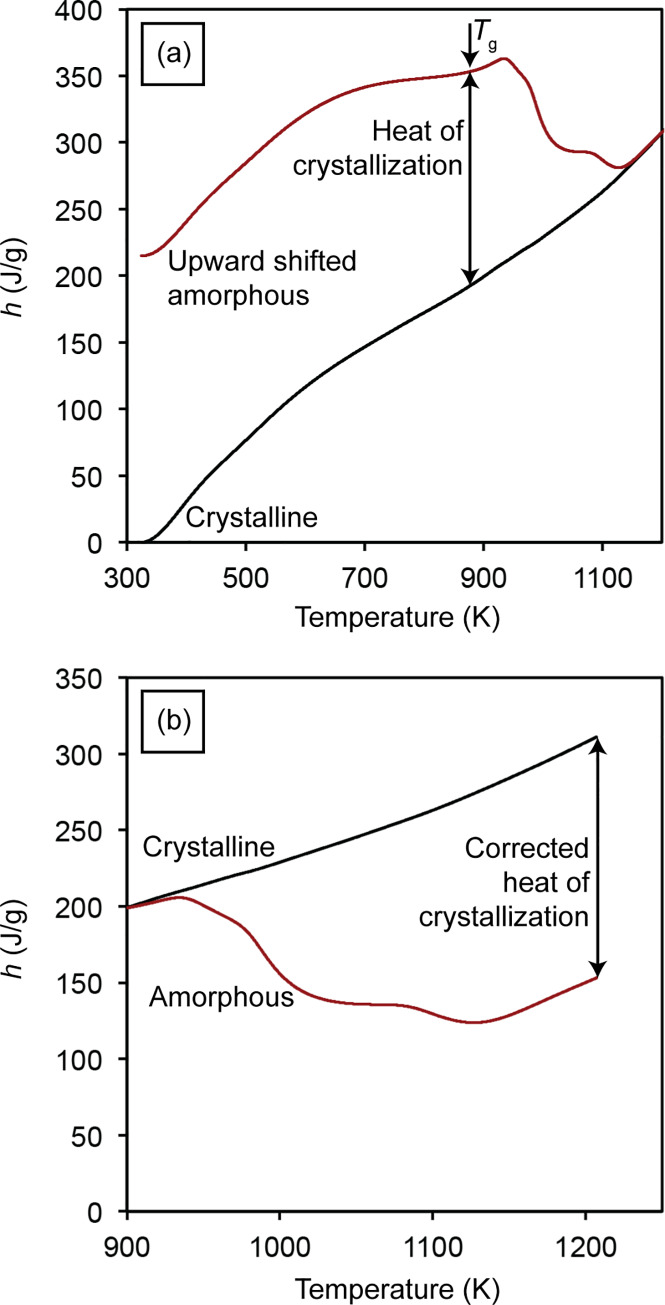
(a) Upward-shifted enthalpy curve of the amorphous powders with respect to the enthalpy curve of the crystalline powders and (b) downward-shifted enthalpy curve of the crystalline powders with respect to the enthalpy curve of the amorphous powders.

In order to finalize the analysis, the next step is to move the enthalpy curve of the amorphous SAM2×5 powders upwards to fit that of the crystalline powders at 1173 K [Fig 11(A)]. This is necessary because after crystallization is completed, both samples are ideally the same (*i*.*e*., both are fully crystalline), and the enthalpy curves for both samples above the completion of crystallization (above 1173 K) match each other, as seen in Fig 11(A). During heating of the sample, there are two temperatures at which the slope of the enthalpy curve of the amorphous sample changes. The first one happens at around 573 K, which indicates the initiation of structural relaxation, and the second one occurs at around 883 K, which is the glass transition temperature. The difference between the enthalpy values of the amorphous and crystalline SAM2×5 samples at *T*_g_ indicates the true heat of crystallization, marked in Fig 11(A) with a two-sided arrow. However, for a more straightforward calculation, it is possible to move the original enthalpy curve of the amorphous SAM2×5 sample, shown in Fig 11(A), downward until it matches that of the crystalline SAM2×5 sample at *T*_g_. We set this point at 898 K, slightly below 918 K, as illustrated in Fig 11(B). We can safely ignore the enthalpy curves below 898 K, as justified earlier, and consider the region beyond that for further calculations. In this case, the difference between the enthalpy curves of the amorphous and crystalline SAM2×5 samples at the maximum temperature indicates the corrected heat of crystallization of the amorphous SAM2×5, which is a constant value. By dividing the difference between the enthalpy of the amorphous and crystalline SAM2×5 samples, at any arbitrary temperature, by the corrected heat of crystallization (a constant), the change of crystallinity of the amorphous SAM2×5 as a function of temperature can be determined. As the temperature of the amorphous sample is increased and reaches the maximum temperature, it becomes fully crystalline, resulting in a change of crystallinity of 1 (or 100% for crystallinity percentage).

**Fig 12:**
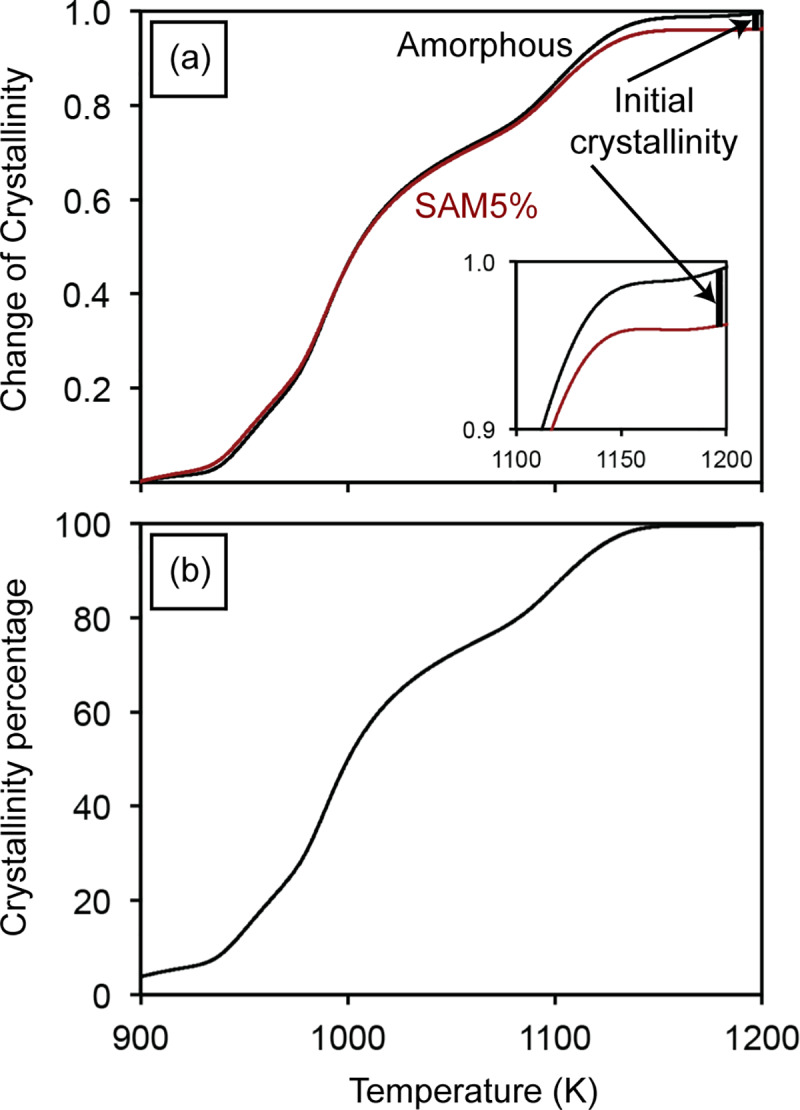
(a) Change of crystallinity of amorphous and SAM5% powders and (b) crystallinity percentage of SAM5% powders as a function of temperature.

Implementing for the SAM5% sample, the change of crystallinity (CC) of the amorphous SAM2×5 and SAM5% samples can be obtained, as illustrated in Fig 12(A) using the following equation:

$$CC\left(T\right)=\frac{h_{crystalline}\left(T\right)-h_{SAMX\%}\left(T\right)}{h_{crystalline}\left(T_{max}\right)-h_{amorphous}\left(T_{max}\right)}$$ (10)

where *h*_SAMX%_ would correspond to the enthalpy for the SAM5% sample (*i*.*e*., *h*_SAM5%_). The initial crystallinity of SAM5% would be the difference between change of crystallinity of the amorphous SAM2×5 and SAM5% powders at the maximum temperature. This process results in a value of approximately 0.04 for one experiment on the SAM5% powders. Moreover, by adding this initial crystallinity value to the change of crystallinity curve at all temperatures for SAM5%, the crystallinity percentage as a function of temperature can be obtained [Fig 12(B)]:

CrystallinityPercentage=[CC(T)+InitialCrystallinity]•100 (11)

For the case of the SAM5% powders, the sample starts with a crystallinity of 4% (*i*.*e*., this is the initial crystallinity). By increasing the temperature to about 1173 K, the crystallinity percentage gradually increases and reaches 100%. Further increases in temperature do not change the crystallinity, as evidenced by the flattening of the curve in Fig 12(B).

**Fig 13:**
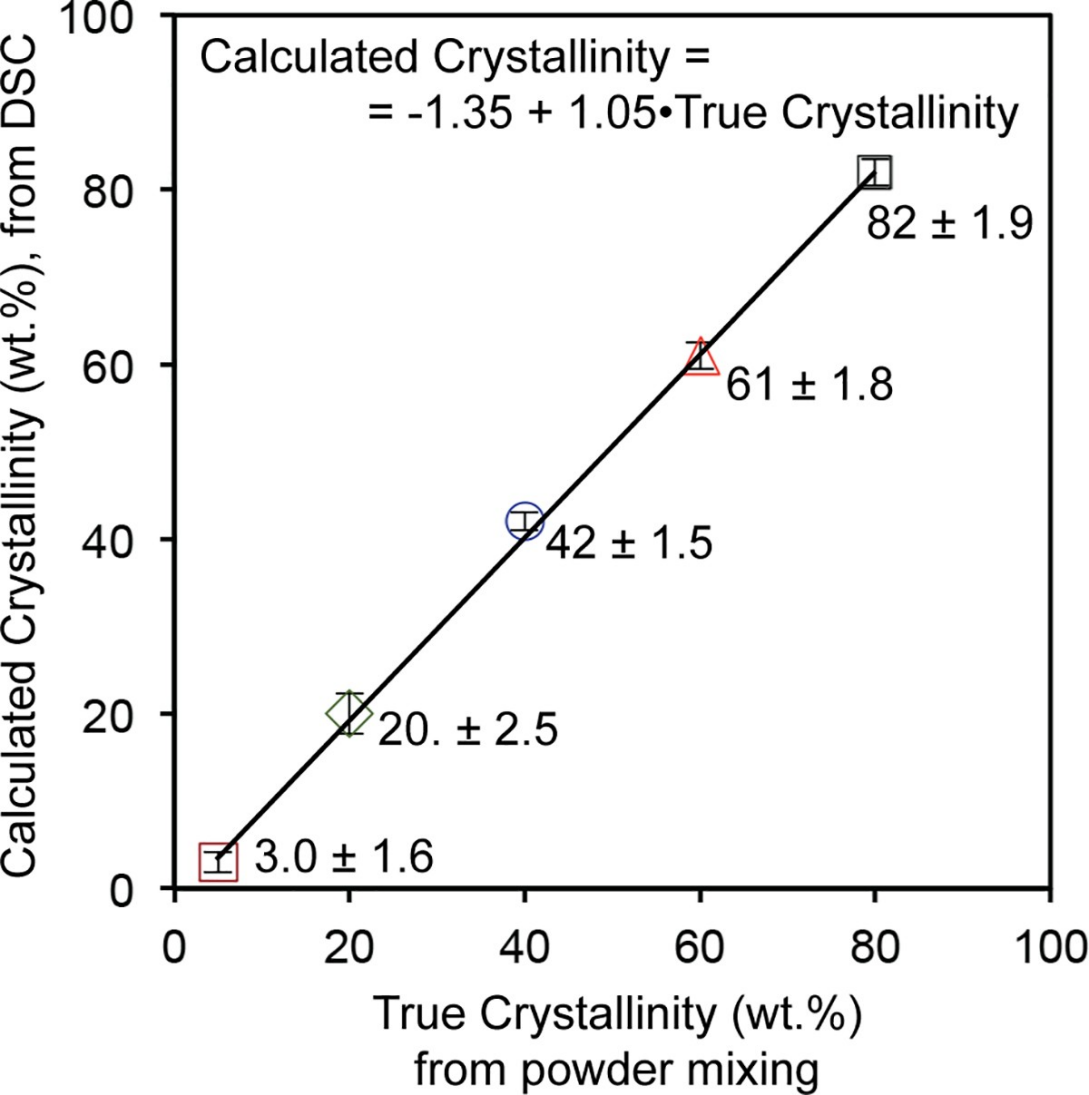
Correlation between calculated crystallinity from DSC results and the true values based on initial known crystallinities of mixed powders.

Crystallinity percentages of powder mixtures of SAM20%, SAM40%, SAM60% and SAM80% were calculated similarly and the results are graphed in Fig 13. Error bars along the *y* axis represent uncertainties that will be discussed in the next section. There is a linear relationship, with a slope close to 1 and intercept of almost zero, between the crystallinity percentage calculated from DSC data and the true crystallinity based on the initial powder mixtures. In other words, the crystallinity values calculated from DSC data equal that of known samples and consequently there is no need to obtain calibration curves of any kind. One only needs an amorphous sample, a crystalline sample, and the test sample. This is one of the merits of our DSC-based methodology. The technique eliminates the need for preparing calibration samples (with known crystallinity percentages) [39].

**Fig 14:**
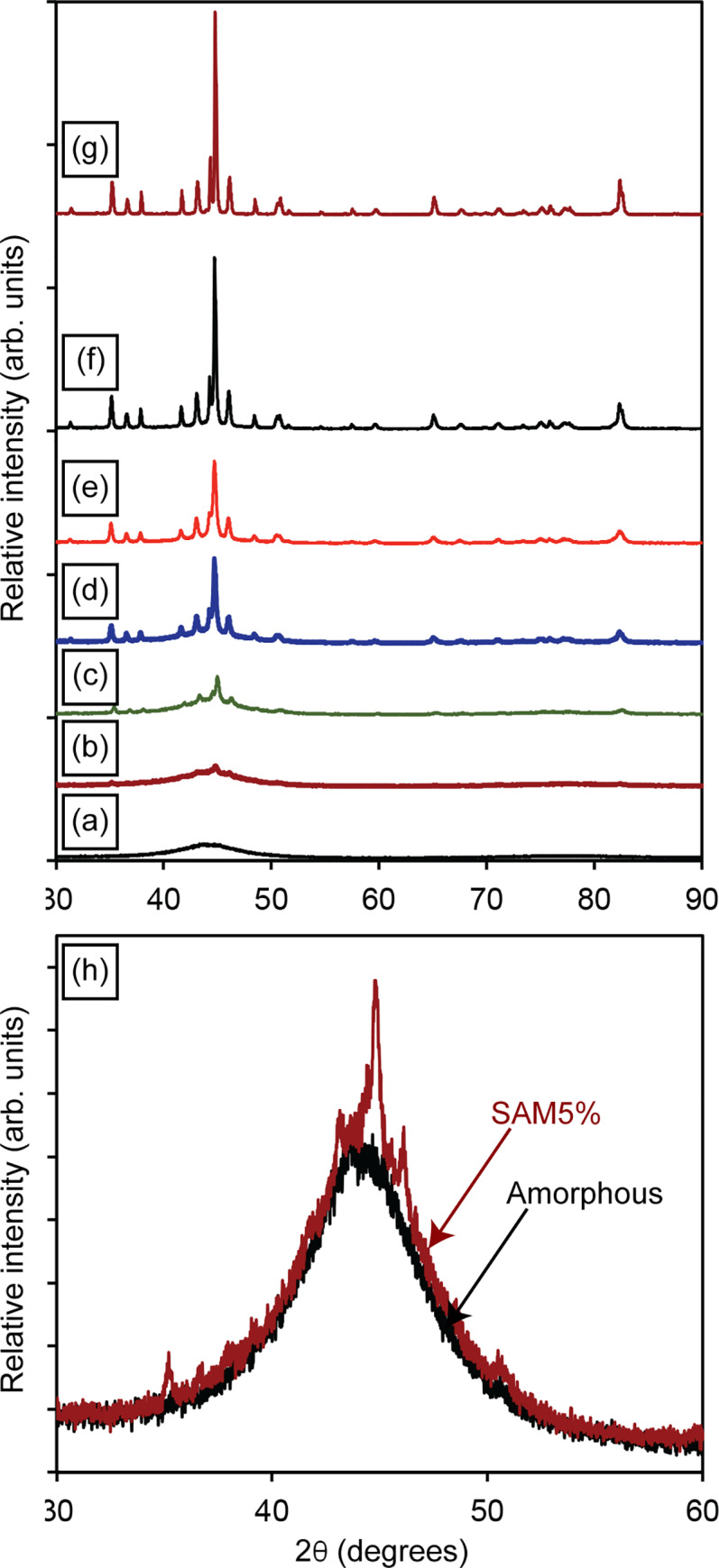
XRD patterns of (a) amorphous, (b) SAM5%, (c) SAM20%, (d) SAM40%, (e) SAM60%, (f) SAM80%, and (g) crystalline powders. (h) Truncated view of XRD pattern of amorphous and SAM5% powders over the 2θ range of 30 to 60 degrees.

Fig 14(A)–14(G) shows the increasing crystallinity of prepared powder mixtures. A truncated view of the XRD patterns of the amorphous SAM2×5 and SAM5% powders in a 2θ range between 30 and 60 degrees is illustrated in Fig 14(H). Despite using a very slow scan rate in XRD, the patterns of amorphous SAM2×5 and SAM5% are very similar, thus it is impossible to quantify the crystallinity of SAM5% via XRD methods. This limitation is expected since the resolution of the XRD technique is about 5 wt.% for specimens without light-weight elements such as carbon and boron [72]. Lack of accuracy of XRD quantification becomes more significant for SAM2×5 materials, which have both boron and carbon at relatively high concentrations. Difficulty in identifying the devitrified phases in SAM2×5 is another reason that precludes XRD as a technique for studying the quantification of crystallization of SAM2×5. However, DSC can overcome the XRD limitations and yield a crystallinity percentage of 3.0 ± 1.6% for SAM5%. We clarify that the crystallinity percentage of 4% obtained for the SAM5% powder [Fig 12(A)] is only from one measurement. However, the numbers reported in Fig 13 are average values determined from three independent measurements. Thus, resulting in a more accurate value that now also contains a standard error.

**Fig 15:**
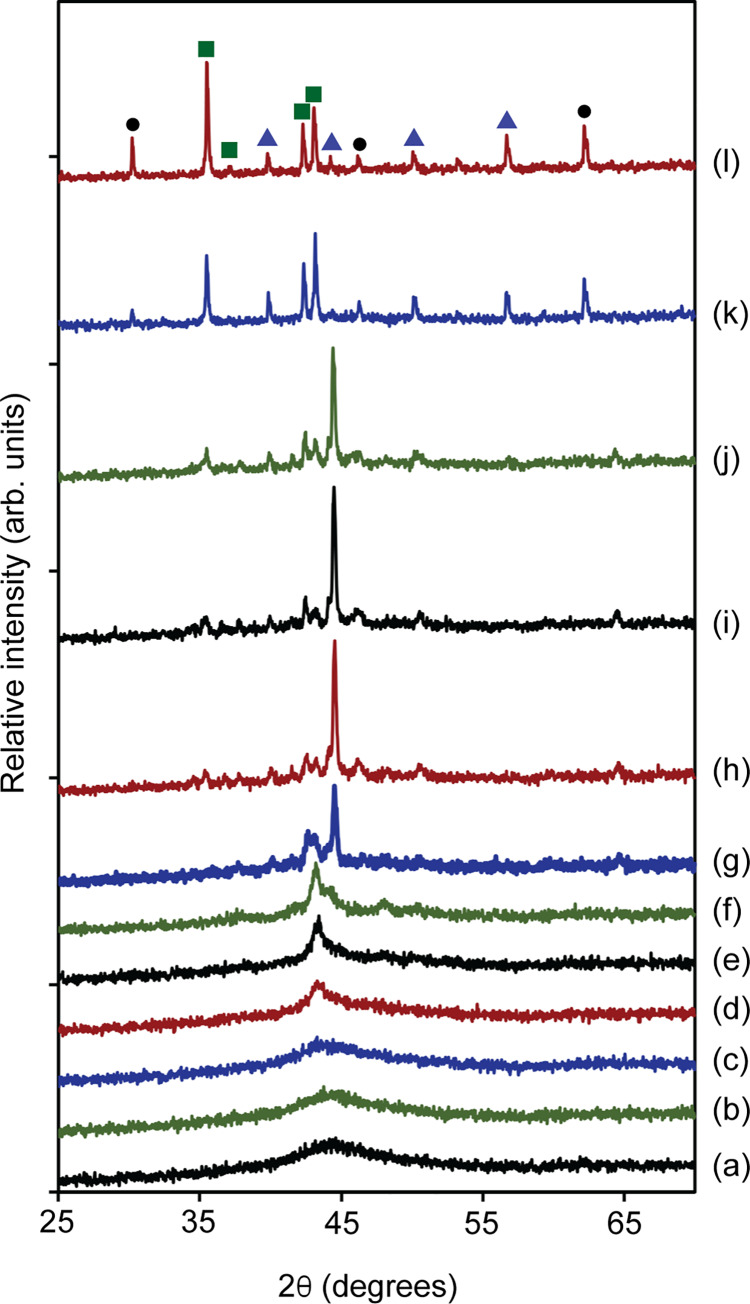
High temperature *in situ* X-ray diffraction patterns of SAM2×5 powders in the temperature range from 823 to 1273 K. The temperature at which each pattern was obtained is (a) 823 K, (b) 848 K, (c) 873 K, (d) 898 K, (e) 923 K, (f) 973 K, (g) 1023 K, (h) 1073 K, (i) 1123 K, (j) 1173 K, (k) 1223 K, and (l) 1273 K. Indexing symbols correspond to those used in Fig 3.

In order to confirm that the exothermic signals observed in DSC curves belong to the crystallization events and not phase transformations from one crystalline state to another, high temperature, *in situ* XRD experiments were completed on the amorphous SAM2×5 powder from 823 to 1273 K. The results are illustrated in Fig 15. At all temperatures below *T*_g_ (883 K), there is no sign of crystallization. At a temperature of 898 K, which is between *T*_g_ and the temperature of initial crystallization, peak splitting begins, indicating initiation of devitrification. Iron is the first phase that crystallizes. At around 1023 K the crystallization of carbide and boride phases then follows. Although experiments were conducted in a hydrogen atmosphere, at a temperature around 1023 K, oxides also start to form, and the quantities increase as the temperature increases, thus the powders do contain some oxygen. This, however, does not impact any of the results from our DSC analysis.

In summary, an experimental DSC-based methodology using a non-isothermal approach was developed to determine the initial crystallization, change of crystallinity, and crystallinity percentage of an amorphous metallic alloy as a function of temperature. Regardless of devitrified phases, constituent elements and crystallization temperature range, this novel technique is capable of pinpointing the crystallinity at an arbitrary temperature with a high accuracy. The only significant limitation is a situation in which either an endothermic or exothermic thermal event takes place during the crystallization temperature range.

## 4. Uncertainty analysis

The uncertainty of the crystallinity percentage was calculated according to the Guide to the Expression of Uncertainty in Measurements (GUM) [73]. It should be noted that the uncertainty of the crystallinity percentage is a function of temperature, since some of the contributors to the uncertainty are temperature dependent. In this section, we focus on obtaining the uncertainty of the crystallinity percentage at room temperature (initial crystallinity). However, the same procedure could be implemented for calculation of the uncertainty of the crystallinity percentage at any arbitrary temperature. We consider the three following assumptions: (i) crystallinity remains unchanged in the temperature region below 898 K, (ii) heating rate has been selected in such a way that crystallization takes place at temperatures between 898 K and 1208 K, and (iii) the crystallinity of all samples is assumed to be 100% when heated above 1208 K. Considering these assumptions, the initial crystallinity can be calculated as follows:

InitialCrystallinity(%)=[1−changeofcrystallinity(CC)at1208K]•100 (12)

Also, according to Eq 10, we have:

$$CC\left(1208\;K\right)=\frac{h_{crystalline}\left(1208\;K\right)-h_{sample}\left(1208\;K\right)}{h_{crystalline}\left(1208\;K\right)-h_{amorphous}\left(1208\;K\right)}$$ (13)

Four contributors to the uncertainty of the crystallinity percentage can be identified: (i) repeatability, instrumental errors on the (ii) enthalpy of crystalline SAM2×5 at 1208 K, (iii) enthalpy of amorphous SAM2×5 at 1208 K, and (iv) enthalpy of test sample at 1208 K.

Errors in repeatability could be quantified by calculating the standard deviation of the crystallinity percentage for each sample using:

$$S=\sqrt{\frac{\sum_{i=1}^{n}{\left(x_{i}-\bar{x}\right)}^{2}}{n-1}}$$ (14)

where *x*_i_, $\bar{x}$ and *n* represent the measured values, the average value of all the measurements, and the number of measurements, respectively. The resulting error values are shown as error bars in Fig 13. According to

$$h\left(T\right)-h\left(898\;K\right)=\int_{898\;K}^{T}C_{p}\left(T\right)\;dT≅\sum_{n}C_{p,n}\left(T_{n}\right)\cdot \Delta T$$ (15)

the enthalpy at any arbitrary temperature is a function of heat capacity as well as temperature. Thus, the following approach can be implemented in order to calculate the uncertainty in the enthalpy. When any arbitrary measured quantity Y is not measured directly, but is determined from N quantities X_1_, X_2_,…, X_N_, we have:

$$Y=f\left(X_{1},X_{2},\ldots ,X_{N}\right)$$ (16)

The expectation of each X_*i*_, where *i* denotes value 1, 2,…, N) is denoted by *x*_*i*_. The standard uncertainty of *y*, where *y* is the estimate of the measured Y is obtained by appropriately combining the standard uncertainties of the input estimates *x*_1_, *x*_2_, …, *x*_N_. When the input quantities are correlated, the appropriate expression for the combined uncertainty *u*(*y*) is:

$$u\left(y\right)=\sqrt{\sum_{i=1}^{N}\sum_{j=1}^{N}\frac{\partial f}{\partial x_{i}}\frac{\partial f}{\partial x_{j}}u\left(x_{i},x_{j}\right)}=\sqrt{\sum_{i=1}^{N}{\left(\frac{\partial f}{\partial x_{i}}\right)}^{2}u^{2}\left(x_{i}\right)+2\sum_{i=1}^{N-1}\sum_{j=i+1}^{N}\frac{\partial f}{\partial x_{i}}\frac{\partial f}{\partial x_{j}}u\left(x_{i},x_{j}\right)}$$ (17)

where *x*_*i*_ and *x*_*j*_ are the estimates of X_*i*_ and X_*j*_ and *u*(*x*_*i*_, *x*_*j*_) = *u*(*x*_*j*_, *x*_*i*_) is the estimated covariance associated with *x*_*i*_ and *x*_*j*_. The degree of correlation between *x*_*i*_ and *x*_*j*_ is characterized by the estimated correlation coefficient:

$$r\left(x_{i},x_{j}\right)=\frac{u\left(x_{i},x_{j}\right)}{u\left(x_{i}\right)\cdot u\left(x_{j}\right)}$$ (18)

where *r*(*x*_*i*_, *x*_*j*_) = *r*(*x*_*j*_, *x*_*i*_) and -1 ≤ *r*(*x*_*i*_, *x*_*j*_) ≤ 1. If the estimates *x*_*i*_ and *x*_*j*_ are independent, then *r*(*x*_*i*_, *x*_*j*_) = 0, and a change in one does not imply an expected change in the other.

From Eq 15, the uncertainty in the enthalpy is affected by the uncertainty of the temperature difference (Δ*T*) and of the heat capacity. Considering the three enthalpy parameters in Eq 13, we start by calculating the uncertainty of *h*_cryst_ (*T* = 1208 K) that, according to Eq 17, could be defined as follows:

$$u\left(h_{cryst}\left(1208\;K\right)\right)=\sqrt{\begin{array}{l} \sum_{i=1}^{n}{\left(\frac{\partial h\left(1208\;K\right)}{\partial x_{i}}\right)}^{2}\cdot u{\left(x_{i}\right)}^{2}+\\ \;+2\sum_{i=1}^{n-1}\sum_{j=i+1}^{n}\left(\frac{\partial h\left(1208\;K\right)}{\partial x_{i}}\right)\left(\frac{\partial h\left(1208\;K\right)}{\partial x_{j}}\right)\cdot u\left(x_{i},x_{j}\right) \end{array}}$$ (19)

where *n* = 3, namely *C*_p-crystalline_sample_ (1208 K) = 0.5561 J/g (average value of all three measurements), *C*_p-crystalline_sample_ (898 K) = 0.35367 J/g (average value of all three measurements), and Δ*T* = 1208–898 = 310 K. Also, *x*_*i*_ and *x*_*j*_ represent different heat capacity values and *u*(*x*_*i*_) is the uncertainty from each input.

The heat capacity values of a specific sample at any two arbitrary temperatures, such as 898 K and 1208 K, are correlated input quantities. Consequently, correlation coefficients need to be considered and calculated. In this research, an online calculator [74] was used to obtain the correlation coefficients. By expanding Eq 19, we obtain:

$$\begin{array}{l} u\left(h_{cryst}\left(T=1208\;K\right)\right)=\\ \;={\left\{\begin{array}{l} {\left(\frac{\partial h\left(1208\;K\right)}{\partial C_{p}\left(898\;K\right)}\right)}^{2}\cdot u{\left(C_{p}\left(898\;K\right)\right)}^{2}+\\ \;+{\left(\frac{\partial h\left(1208\;K\right)}{\partial C_{p}\left(1208\;K\right)}\right)}^{2}\cdot u{\left(C_{p}\left(1208\;K\right)\right)}^{2}+\\ \;+{\left(\frac{\partial h\left(1208\;K\right)}{\partial \Delta T}\right)}^{2}\cdot u{\left(\Delta T\right)}^{2}+\\ \;+2\cdot \left[\begin{array}{l} \left(\frac{\partial h\left(1208\;K\right)}{\partial C_{p}\left(898\;K\right)}\right)\cdot \left(\frac{\partial h\left(1208\;K\right)}{\partial C_{p}\left(1208\;K\right)}\right)\cdot \\ r\left(C_{p}\left(898\;K\right),C_{p}\left(1208\;K\right)\right)\cdot \\ u\left(C_{p}\left(898\;K\right)\right)\cdot u\left(C_{p}\left(1208\;K\right)\right)+\\ \left(\frac{\partial h\left(1208\;K\right)}{\partial C_{p}\left(898\;K\right)}\right)\cdot \left(\frac{\partial h\left(1208\;K\right)}{\partial \Delta T}\right)\cdot \\ r\left(C_{p}\left(898\;K\right),\Delta T\right)\cdot u\left(C_{p}\left(898\;K\right)\right)\cdot u\left(\Delta T\right)+\\ \left(\frac{\partial h\left(1208\;K\right)}{\partial C_{p}\left(1208\;K\right)}\right)\cdot \left(\frac{\partial h\left(1208\;K\right)}{\partial \Delta T}\right)\cdot \\ r\left(C_{p}\left(1208\;K\right),\Delta T\right)\cdot u\left(C_{p}\left(1208\;K\right)\right)\cdot u\left(\Delta T\right) \end{array}\right] \end{array}\right\}}^{\frac{1}{2}} \end{array}$$ (20)

In order to calculate the uncertainty of heat capacity at 898 K, *u*(*C*_p_(898 K)), and 1208 K, *u*(*C*_p_(1208 K)), instead of calculating the standard deviation, the difference between the maximum and minimum values of heat capacity at 898 K and 1208 K are considered. This is for the purpose of incorporating larger values of the uncertainty of the heat capacity, which yields a larger safety factor for the total uncertainty. The uncertainty of the temperature difference (Δ*T*) of 1 K (based on information from TA Instruments), is an expanded uncertainty with a coverage factor of 2. Since the heat capacities at 898 K and 1208 K are independent of the temperature difference (Δ*T*), the corresponding correlation coefficients *r*(*C*_p_(898 K)) and *r*(*C*_p_(1208 K)) are zero, meaning that the last two terms in the bracket in Eq 20 are equal to zero. However, from the online calculator, the correlation coefficient between heat capacities was calculated to be 0.92576. Consequently, by inserting the corresponding values into Eq 20, we obtain:

$$\begin{array}{l} u\left(h_{cryst}\left(T=1208\;K\right)\right)=\\ \;={\left\{\begin{array}{l} {\left(\Delta T\right)}^{2}\cdot u{\left(C_{p}\left(898\;K\right)\right)}^{2}+{\left(\Delta T\right)}^{2}\cdot u{\left(C_{p}\left(1208\;K\right)\right)}^{2}+\\ \;+{\left(C_{p}\left(898\;K\right)+C_{p}\left(1208\;K\right)\right)}^{2}\cdot u{\left(\Delta T\right)}^{2}+\\ \;+2\left[\begin{array}{l} {\left(\Delta T\right)}^{2}\cdot r\left(C_{p}\left(898\;K\right),C_{p}\left(1208\;K\right)\right)\cdot \\ u\left(C_{p}\left(898\;K\right)\right)\cdot u\left(C_{p}\left(1208\;K\right)\right) \end{array}\right] \end{array}\right\}}^{\frac{1}{2}}=\\ \;={\left\{\begin{array}{l} {\left(310\right)}^{2}\cdot {\left(0.1878\right)}^{2}+{\left(310\right)}^{2}\cdot {\left(0.3118\right)}^{2}+\\ \;+{\left(0.5661+0.35367\right)}^{2}\cdot {\left(0.05\right)}^{2}+\\ \;+2\left[{\left(310\right)}^{2}\cdot \left(0.92576\right)\cdot \left(0.1878\right)\cdot \left(0.3118\right)\right] \end{array}\right\}}^{\frac{1}{2}}=152.15\;J/g \end{array}$$ (21)

Similarly, *u*(*h*_amorphous_(T = 1208 K)) was calculated and resulted in a value of 91.21 J/g. The same procedure was repeated for SAM5% and *u*(*h*_SAM5%_(T = 1208 K)) was found to be 69.14 J/g. According to GUM, uncertainty contributions must all be in the same units of measurement before they can be combined. In order to do that, sensitivity coefficients for each contributor to uncertainty need to be calculated. Generally, sensitivity coefficients are just a multiplier used to convert the uncertainty components to the correct units and magnitude for uncertainty analysis. A sensitivity coefficient for each input was separately calculated by obtaining the derivative of Eq 13 with respect to the input. Thus, the sensitivity coefficients are:

$$\begin{array}{l} SC\left[h_{crystalline}\left(T=1208\;K\right)\right]=\frac{\partial CC\left(1208\;K\right)}{\partial h_{crystalline}\left(T=1208\;K\right)}=\\ \;=\frac{h_{SAM5\%}\left(T=1208\;K\right)-h_{amorphous}\left(T=1208\;K\right)}{{\left[h_{crystalline}\left(T=1208\;K\right)-h_{amorphous}\left(T=1208\;K\right)\right]}^{2}} \end{array}$$ (22)

$$\begin{array}{l} SC\left[h_{SAM5\%}\left(T=1208\;K\right)\right]=\frac{\partial CC\left(1208\;K\right)}{\partial h_{SAM5\%}\left(T=1208\;K\right)}=\\ \;=\frac{1}{h_{amorphous}\left(T=1208\;K\right)-h_{crystalline}\left(T=1208\;K\right)} \end{array}$$ (23)

$$\begin{array}{l} SC\left[h_{amorphous}\left(T=1208\;K\right)\right]=\frac{\partial CC\left(1208\;K\right)}{\partial h_{amorphous}\left(T=1208\;K\right)}=\\ \;=\frac{h_{crystalline}\left(T=1208\;K\right)-h_{SAM5\%}\left(T=1208\;K\right)}{{\left[h_{crystalline}\left(T=1208\;K\right)-h_{amorphous}\left(T=1208\;K\right)\right]}^{2}} \end{array}$$ (24)

Considering the average values for *h*_crystalline_(*T* = 1208 K), *h*_SAM5%_(*T* = 1208 K) and *h*_amorphous_(*T* = 1208 K) of 344.47, 119.68 and 125.97, respectively, the sensitivity coefficient for each enthalpy factor can be calculated. Since the units of repeatability and enthalpy are similar, the sensitivity coefficient for repeatability is assumed to be 1. Contributors to uncertainty and sensitivity coefficients have been listed in Table 2. Finally, in order to combine the contributions, the following equation was used:

$$u_{c}=\sqrt{\sum_{i}c_{i}^{2}u_{i}^{2}}$$ (25)

where *u*_*c*_, *c*_i_^2^ and *u*_*i*_^2^ are the total (combined uncertainty), sensitivity coefficient for factor *i* and uncertainty regarding factor *i*, respectively. Using Eq 25 and data in Table 2, the uncertainty of the crystallinity percent of a powder mixture with a known crystallinity of 5% is 1.6%. Aside from the sources of uncertainty contributing to the calculated crystallinity, it should be noted that the process of preparing calibration samples with known crystallinity also contributes to the error. Two main contributors to the uncertainty of crystallinity of calibration samples include (i) measuring the weight of powder samples, and (ii) repeatability. For preparing calibration samples (contribution i), appropriate amounts of crystalline and amorphous SAM2×5 were weighted with a repeatability of 0.2 g (provided by the manufacturer of the scale). Since two mass measurements were carried out for preparing each calibration sample, the theoretical uncertainty would be:

$$\sqrt{{\left(0.2\;g\right)}^{2}+{\left(0.2\;g\right)}^{2}}=0.28\;g$$ (26)

For each crystallinity percentage, three powder mixtures were independently prepared and tested, thus the contribution of repeatability could be quantified in the form of a standard deviation presented in the last column of Table 1 (contribution ii). As mentioned earlier, the sensitivity coefficient for these two types of repeatability equals to 1. Hence, using Eq 25, the rounded combined uncertainties for the crystallinity percentage of each prepared powder mixture are calculated and presented in Table 3. The uncertainty values associated with the crystallinity of calibration samples determined from experiments and calculation are listed in Table 4 and as error bars along the *y* axis in Fig 13. Table 4 summarizes the true and calculated crystallinities and corresponding uncertainties for all calibration specimens. It is clearly seen that for all concentrations of crystalline phase in the calibration samples, the calculated crystallinity is very close to the true one. This high accuracy is even observed for the sample with an extreme composition (SAM5%). It is worth mentioning that ideally assumed amorphous samples might include some small percentage of crystalline phase, while the crystalline samples might include some small percentage of amorphous component. This would need to be quantified and added to the uncertainty associated with the preparation of calibration samples.

**Table 2:** Uncertainty and sensitivity coefficients.

Uncertainty source	Uncertainty	Units	Sensitivity coefficient
Repeatability	1.15	wt.%	1
*h*_crystalline_(*T* = 1208 K)	152.15	J/g	-0.000129236
*h*_amorphous_(*T* = 1208 K)	91.21	J/g	0.004705895
*h*_SAM5%_(*T* = 1208 K)	69.14	J/g	-0.004576659

**Table 3:** Uncertainties of sample weighting and repeatability, combined uncertainties, and rounded combined uncertainties for calibration samples with different crystallinity percentages.

Sample	Uncertainty of sample weighting	Uncertainty of repeatability	Combined uncertainty	Rounded combined uncertainty
SAM5%	0.28	0.1	[(0.28)^2^ +(0.1)^2^]^0.5^ = 0.297	0.30
SAM20%	0.28	0.1	[(0.28)^2^ + (0.1)^2^]^0.5^ = 0.297	0.30
SAM40%	0.28	0.2	[(0.28)^2^ + (0.2)^2^]^0.5^ = 0.3440	0.30
SAM60%	0.28	0.1	[(0.28)^2^ +(0.1)^2^]^0.5^ = 0.297	0.30
SAM80%	0.28	0.1	[(0.28)^2^ +(0.1)^2^]^0.5^ = 0.297	0.30

**Table 4:** Calculated crystallinity and uncertainty for powder mixtures with known crystallinity percentages. The values following the average crystallinity in the last column of Table 1 include the standard deviation. The errors in the initial crystallinity in this table are uncertainty values that include the standard deviation obtained from three independent measurements as well as errors originating from multiple sources explained in the manuscript.

Sample	Values from powder mixtures		Values from DSC determination	
	Known crystallinity (wt.%)	Uncertainty (wt.%)	Calculated crystallinity from DSC data (wt.%)	Uncertainty (wt.%)
SAM5%	5.1	0.3	3.0	1.6
SAM20%	20.0	0.3	20.0	2.5
SAM40%	40.0	0.3	42.0	1.5
SAM60%	60.0	0.3	61.0	1.8
SAM80%	80.0	0.3	82.0	1.9

## 5. Conclusions

A novel method based on differential scanning calorimetry (DSC) is introduced for the determination of initial crystallinity, change of crystallinity, and crystallinity percentage as a function of temperature for amorphous metal alloys by comparing the enthalpy of amorphous, crystalline and partially crystalline specimens. In order to ascertain that the values of calculated crystallinity are close to those of set crystallinities, five calibration samples with different crystallinities were prepared by mixing appropriate amounts of amorphous and crystalline powders. Due to the nature of the DSC technique, there is no need for homogenous mixing of calibration samples, which is considered a merit of this method compared to other characterization methodologies such as X-ray diffraction (XRD). Through a dynamic DSC approach, the variation of heat capacity and enthalpy of amorphous, crystalline and powder mixtures with a known crystallinity were calculated. A linear relationship between the true and calculated crystallinity values guarantees that, unlike conventional methods such as XRD, it is not necessary to prepare and test calibration samples before examining a target specimen. We also used the GUM methodology to quantify the contributions from random and systematic errors. We found that for the pre-mixed sample with composition of 5% crystalline and 95% amorphous content, our methodology yields a crystallinity of 3.0 ± 1.6%. This methodology has a very high accuracy as well as a very broad applicability to diverse amorphous systems including metals, ceramics, polymers, and alloys regardless of crystallization temperature range, and devitrified products. The method loses its accuracy when analyzing amorphous systems in which the crystallization process is accompanied by the occurrence of other phase transformations at the same temperature. Also, to analyze materials in which melting takes place slightly after crystallization terminates, a significantly lower heating rate needs to be incorporated.
